# Association of bovine major histocompatibility complex (BoLA) gene polymorphism with colostrum and milk microbiota of dairy cows during the first week of lactation

**DOI:** 10.1186/s40168-018-0586-1

**Published:** 2018-11-12

**Authors:** Hooman Derakhshani, Jan C. Plaizier, Jeroen De Buck, Herman W. Barkema, Ehsan Khafipour

**Affiliations:** 1 Department of Animal Science, University of Manitoba, Winnipeg, MB Canada; 2 Department of Production Animal Health, Faculty of Veterinary Medicine, University of Calgary, Calgary, AB Canada; 3 Department of Medical Microbiology and Infectious Diseases, University of Manitoba, 225 Animal Science Bldg., Winnipeg, MB R3T 2N2 Canada

**Keywords:** Bovine major histocompatibility complex (BoLA) gene, Milk microbiota, Mastitis

## Abstract

**Background:**

The interplay between host genotype and commensal microbiota at different body sites can have important implications for health and disease. In dairy cows, polymorphism of bovine major histocompatibility complex (BoLA) gene has been associated with susceptibility to several infectious diseases, most importantly mastitis. However, mechanisms underlying this association are yet poorly understood. In the present study, we sought to explore the association of BoLA gene polymorphism with the dynamics of mammary microbiota during the first week of lactation.

**Results:**

Colostrum and milk samples were collected from multiparous Holstein dairy cows at the day of calving and days 1 and 6 after calving. Microbiota profiling was performed using high-throughput sequencing of the V1-V2 regions of the bacterial 16S rRNA genes and ITS2 region of the fungal ribosomal DNA. Polymorphism of BoLA genes was determined using PCR-RFLP of exon 2 of the *BoLA-DRB3*. In general, transition from colostrum to milk resulted in increased species richness and diversity of both bacterial and fungal communities. The most dominant members of intramammary microbiota included *Staphylococcus*, Ruminococcaceae, and Clostridiales within the bacterial community and *Alternaria*, *Aspergillus*, *Candida*, and *Cryptococcus* within the fungal community. Comparing the composition of intramammary microbiota between identified *BoLA-DRB3.2* variants (*n* = 2) revealed distinct clustering pattern on day 0, whereas this effect was not significant on the microbiota of milk samples collected on subsequent days. On day 0, proportions of several non*-aureus Staphylococcus* (NAS) OTUs, including those aligned to *Staphylococcus equorum*, *Staphylococcus gallinarum*, *Staphylococcus sciuri*, and *Staphylococcus haemolyticus*, were enriched within the microbiota of one of the *BoLA-DRB3.2* variants, whereas lactic acid bacteria (LAB) including *Lactobacillus* and *Enterococcus* were enriched within the colostrum microbiota of the other variant.

**Conclusion:**

Our results suggest a potential role for BoLA-gene polymorphism in modulating the composition of colostrum microbiota in dairy cows. Determining whether BoLA-mediated shifts in the composition of colostrum microbiota are regulated directly by immune system or indirectly by microbiota-derived colonization resistant can have important implications for future development of preventive/therapeutic strategies for controlling mastitis.

**Electronic supplementary material:**

The online version of this article (10.1186/s40168-018-0586-1) contains supplementary material, which is available to authorized users.

## Introduction

Different body sites of vertebrates provide stable and nutrient-rich ecosystems for a diverse range of microbial symbionts to thrive [1, 2]. Microbial symbionts, comprised of different species of bacteria, fungi, and archaea, in turn perform a number of functions that are essential for host physiology, including digestion of complex carbohydrates, biosynthesis of vitamins, and modulation of immune homeostasis [3, 4]. The relationship between host animal and microbiota, however, is not always beneficial. Indeed, host-associated microbiota usually contain a number of opportunistic members (i.e., pathobionts), which under certain circumstances, such as immunosuppression of the host, are capable of dominating the microbiota and disrupting the immune homeostasis at different body sites [5]. Moreover, at the interface with host immune system, commensal microbes can stimulate the activation of a number of pro-inflammatory responses [4]. In order to cope with this constant immunological challenge while maintaining the equilibrium between pro- and anti-inflammatory responses, the immune system of mammals has adapted specific mechanisms for discriminating between commensals, pathobionts, and strict pathogens [6].

An example of this evolutionary adaptation is the polymorphism of the class II region of the major histocompatibility complex (MHC) gene [7]. This gene encodes highly polymorphic glycoproteins on the surface of the professional antigen-presenting cells (APCs) of the immune system, including macrophages, dendritic cells, and B lymphocytes [8]. The main function of the MHC class II glycoproteins is to facilitate the watchdog role of the immune system in recognition of various antigens and triggering pro- and anti-inflammatory responses accordingly [9]. Following internalization by APCs, exogenous antigens are degraded into smaller peptides that can bind to the antigen-binding groove of the MHC class II glycoproteins. The resulting peptide-MHC complex is then transported to the plasma membrane of the APCs and presented to the receptors of the CD4^+^ helper T lymphocytes [8]. Depending on the nature of the antigens presented, activated T lymphocytes will be differentiated into effector T_H_1, T_H_2, or T_H_17 cells, which can in turn trigger a number of pro- and anti-inflammatory responses [10]. MHC heterozygosity and/or certain allelic variations within MHC gene can influence the diversity of the T lymphocyte receptors and therefore confer resistance to microbial colonization by virtue of their ability to trigger immune responses against a wider range of antigens [7]. Indeed, several studies in humans [11], mice [12, 13], and fish [14] have demonstrated that MHC polymorphism can influence the composition of the microbiota that inhabit different niches of the gastrointestinal tract.

In cattle, similar to other species, the physiologic relevance of MHC gene polymorphism has been traditionally viewed from the perspective of host-pathogen interaction and infectious tolerance. In particular, several studies have focused on exploring the associations of allelic variants of MHC gene family, also known as bovine leukocyte antigens (BoLA) genes, with susceptibility to infectious diseases caused by pathogenic bacteria, viruses, and parasites, among others [15–17]. Particularly, in the context of bovine mastitis, which is the most prevalent and costly disease of dairy cows [18, 19], several investigations have detected associations between certain allelic variants of exon 2 of the *BoLA-DRB3.2* gene and resistance to intramammary infection (IMI) by major mastitis pathogens and/or severity of the inflammatory responses of the mammary gland (MG) [20–24]. Nonetheless, direct and indirect mechanisms by which BoLA gene polymorphism contributes to the immune homeostasis of the MG have not yet been described in detail. Recent metagenomics studies have suggested that in addition to major mastitis pathogens, which usually possess a variety of virulence factors evolved to resist the defense mechanisms of the MG [25, 26], intramammary secretions of dairy cows can harbor a broad range of non-pathogenic bacterial groups [27–32]. However, whether BoLA gene polymorphism can modulate the composition of the intramammary microbiota remains unknown. Indeed, the paucity of evidence for crosstalk between MG microbiota and cows’ genotype has raised concerns regarding the validity of the concept of intramammary commensal microbiota [33]. Hence, the main objective of the present study was to evaluate the potential association of *BoLA*-*DRB3* polymorphism with the composition and dynamics of bacterial and fungal components of the intramammary microbiota of dairy cows during the first week of lactation, coinciding with the transition from colostrum to milk.

## Results

### Identification of *BoLA*-*DRB3.2* variants and sequencing results

Based on the digestion activity of the BstYI enzyme, three distinct *BoLA*-*DRB3.2* variants were identified within the studied population of cows (Additional file 1: Figure S1), named herein BstYa (*n* = 24), BstYb (*n* = 25), and BstYc (*n* = 5). Due to the limited number of cows that were categorized under the BstYc variant, and in order to maintain statistical power of between-variants comparisons, all the colostrum and milk samples belonging to this variant were excluded from downstream microbial analyses. For BstYa and BstYb variants, after quality filtering of the sequencing reads and removing samples with low sequencing depth (< 5000 sequence per sample), a total of 132 and 82 samples were kept for downstream analyses of bacterial and fungal communities. On average, 28,727 (6889–100,740; SD = 13,921) and 35,328 (8657–119,756; SD = 17,175) high-quality 16S rRNA and ITS2 sequences were obtained per sample, resulting in identification of 590 (83–1155; SD = 290) and 24 (6–80; SD = 13) representative bacterial and fungal OTUs, respectively.

### Biodiversity dynamics of intramammary secretions during the first week of lactation: associations with *BoLA*-*DRB3.2* polymorphism

**Fig. 1:**
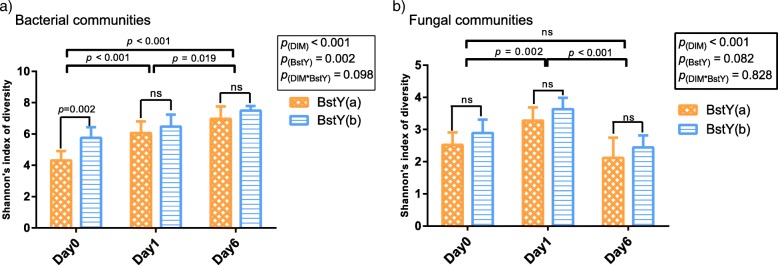
Comparison of the diversity of intramammary microbiota during the first week of lactation. Shannon’s indexes of diversity were compared among **a** bacterial and **b** fungal communities of intramammary secretions. Bacterial and fungal OTU tables were normalized to an even depth of 6000 and 5000 OTUs per sample, respectively, prior to calculation of diversity metrics. Mixed linear model (repeated measurement; proc. mixed SAS) was fitted with days in milk (DIM), BstYI variants, and their interaction as fixed factors and the effect of individual cows as a random factor nested within the BstYI variants. Error bars denote the 95% confidence intervals. For all tests, *p* values < 0.05 were considered significant. “ns” indicates *p* values > 0.05. Summary statistics for all comparisons are available in Additional file 2: Table S1

Overall, diversity of intramammary microbiota (Shannon’s index) underwent significant changes during the first week of lactation (Fig. 1a, b). Diversity of bacterial communities increased over time (*p* < 0.001), with day 6 samples harboring the most diverse communities across all three sampling time points. On the other hand, diversity of fungal communities was higher on day 1 compared to day 0 (*p* = 0.002) and day 6 (*p* < 0.001). The association between *BoLA*-*DRB3.2* polymorphism and the diversity of intramammary microbiota was only significant on day 0, when colostrum samples of BstYb variant harbored bacterial communities that were more diverse than those of the samples belonging to BstYa variant (*p* = 0.002). There was no association between *BoLA*-*DRB3.2* polymorphism and Shannon’s index of diversity of fungal communities (*p* = 0.082; summary statistics for alpha-diversity comparisons available in Additional file 2: Table S1).

**Fig. 2:**
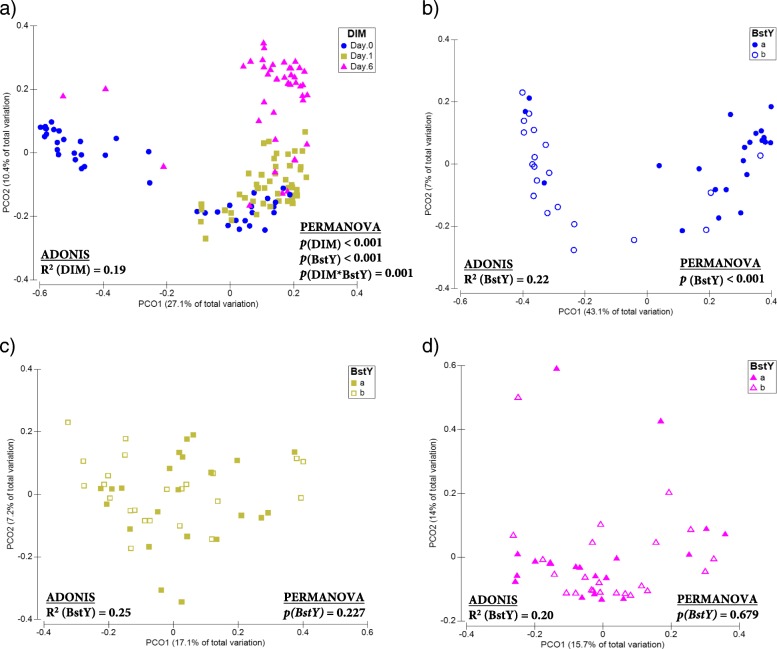
Beta-diversity of the bacterial communities of intramammary secretions during the first week of lactation. Principal coordinate analysis (PCoA) was used for visualization of Bray-Curtis dissimilarities of the bacterial communities of intramammary secretions. Color codes and symbols were used to differentiate samples based on **a** days in milk (DIM), **b** BstYI variants within day 0, **c** BstYI variants within day 1, and **d** BstYI variants within day 6. Prior to calculation of Bray-Curtis dissimilarities, the OTU table was normalized using cumulative sum scaling (CSS) transformation. PERMANOVA (9999 permutations) was performed on a repeated measurement model that included DIM, BstYI variants, and the interaction between DIM and BstYI variants as fixed factors and the effect of individual cows as a random factor nested within the BstYI variants. For all tests, *p* values < 0.05 were considered significant

**Fig. 3:**
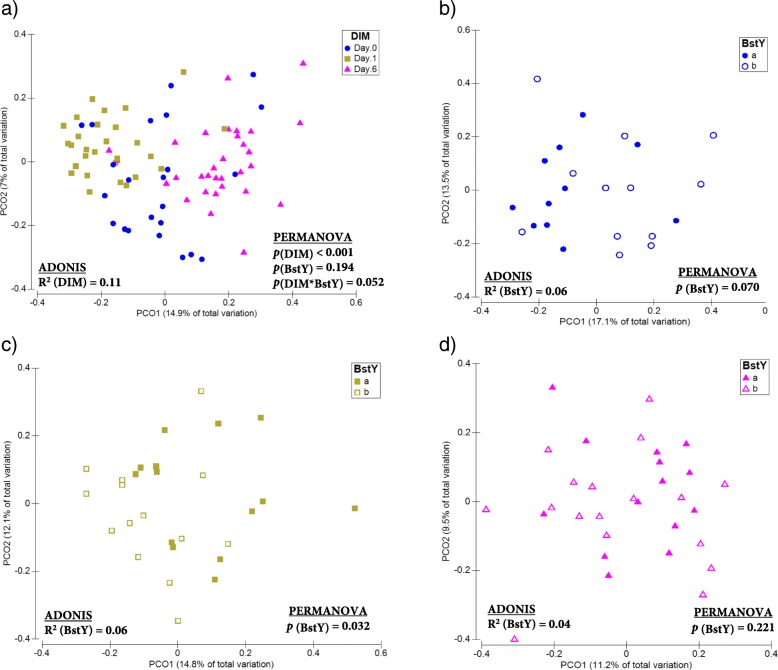
Beta-diversity of the fungal communities of intramammary secretions during the first week of lactation. Principal coordinate analysis (PCoA) was used for visualization of Bray-Curtis dissimilarities of the fungal communities of intramammary secretions. Color codes and symbols were used to differentiate samples based on **a** days in milk (DIM), **b** BstYI variants within day 0, **c** BstYI variants within day 1, and **d** BstYI variants within day 6. Prior to calculation of Bray-Curtis dissimilarities, the OTU table was normalized using cumulative sum scaling (CSS) transformation. PERMANOVA (9999 permutations) was performed on a repeated measurement model that included DIM, BstYI variants, and the interaction between DIM and BstYI variants as fixed factors and the effect of individual cows as a random factor nested within the BstYI variants. For all tests, *p* values < 0.05 were considered significant

PCoA and permutational multivariate analysis of variance (PERMANOVA) of Bray-Curtis dissimilarities revealed that the composition of both bacterial (ADONIS R^2^ = 0.19, *p*_(PERMANOVA)_ < 0.001; Fig. 2a) and fungal (ADONIS *R*^2^ = 0.11, *p*_(PERMANOVA)_ < 0.001; Fig. 3a) communities of intramammary secretions differed among all sampling time points. The association between *BoLA*-*DRB3.2* polymorphism and the composition of bacterial communities was only significant on day 0, when colostrum samples belonging to BstYa and BstYb variants showed distinct clustering pattern (ADONIS *R*^2^ = 0.22, *p*_(PERMANOVA)_ < 0.001; Fig. 2b–d). *BoLA*-*DRB3.2* polymorphism was also associated with the composition of fungal communities of intramammary secretions on day 0 (ADONIS *R*^2^ = 0.06, *p*_(PERMANOVA)_ = 0.070) and day 1 (ADONIS *R*^2^ = 0.06, *p*_(PERMANOVA)_ = 0.032), but not on day 6 (ADONIS *R*^2^ = 0.04, *p*_(PERMANOVA)_ = 0.226; Fig. 3b–d). PCoA and PERMANOVA based on Jaccard binary distances of bacterial and fungal communities also revealed similar clustering patterns based on sampling time points and *BoLA*-*DRB3.2* polymorphism (Additional file 1: Figures S2 and S3). Summary statistics for PERMANOVA analyses are presented in Additional file 2: Table S2.

### Dynamics of intramammary microbiota in relation to days in milk and *BoLA*-*DRB3.2* polymorphism

**Fig. 4:**
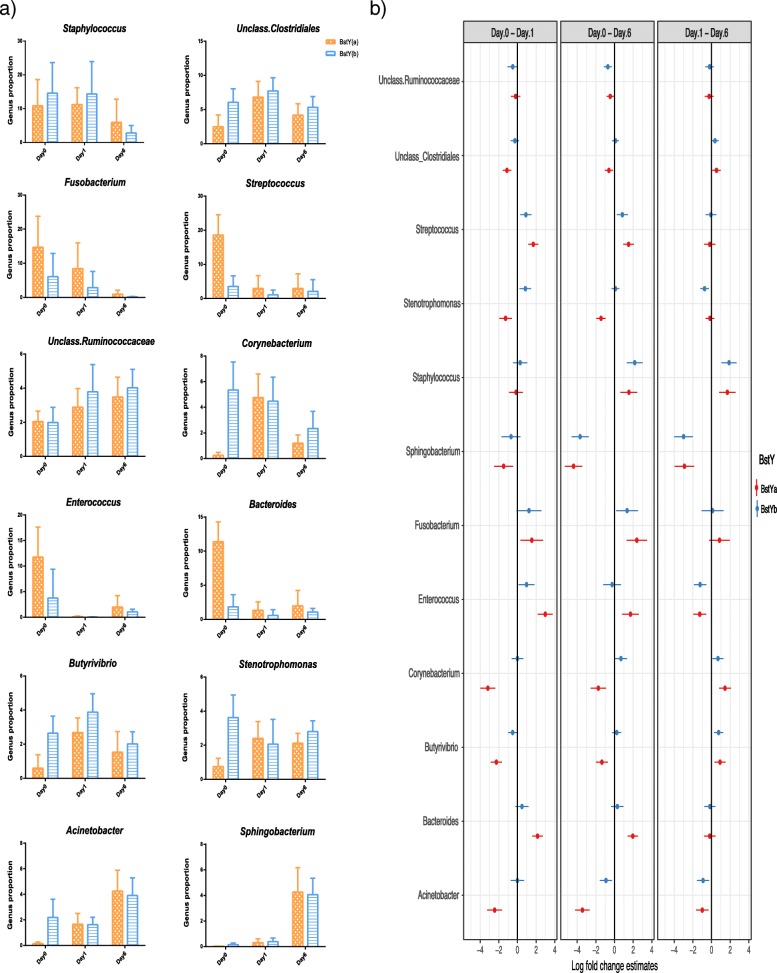
Dynamics of bacterial communities of intramammary microbiota during the first week of lactation. **a** Bar graphs show the proportion of predominant bacterial genera of intramammary secretions during the first week of lactation. Color codes were used to relate the average proportion of genera to BoLA variants, *x*-axis relates the proportion of bacterial genera to days in milk, and error bars denote 95% confidence intervals. **b** Associations of bacterial genera with BoLA variants and DIM were analyzed with generalized linear mixed effect model (package glmmTMB). The total count of OTUs assigned to each genus were offset to the library size (total count of OTUs detected in each sample) and then used as the response variable in a negative binomial model where BoLA variants, DIM, and their interaction were included as fixed effects whereas the effect of individual cows were included as the random effect. Estimated group means, confidence intervals (CI), and pairwise comparisons for effects of BstY variants and DIM were derived using the package emmeans. Multiple hypotheses were adjusted by Benjamini and Hochberg false discovery rate (FDR). Genera for which the high and low values of CI do not cross the zero line shows significant log fold change between DIM contrasts. Summary statistics are available in Additional file 2: Table S3

The bacterial communities of colostrum and milk samples were mainly composed of the members of phyla Firmicutes, Proteobacteria, Bacteroidetes, Actinobacteria, and Fusobacteria. Dominant bacterial genera of mammary microbiota had different trajectory patterns over the first week of lactation and in relation to BoLA variants (Fig. 4a, b). The proportion of *Staphylococcus*, the predominant genus within the colostrum microbiota of both BstY variants, decreased during transition from colostrum to milk. Similarly, proportions of *Streptococcus*, *Fusobacterium*, *Enterococcus*, and *Bacteroides*, which were all overrepresented within the microbiota of BstYa variant on day 0, decreased significantly during the first week of lactation and became proportionally similar between the microbiota of the two BoLA variants on day 6. On the other hand, proportions of *Sphingobacterium* and *Acinetobacter* increased during transition from colostrum to milk within the microbiota of the two BoLA variants. Summary statistics for all pairwise contrasts are presented in Additional file 2: Table S3.

**Fig. 5:**
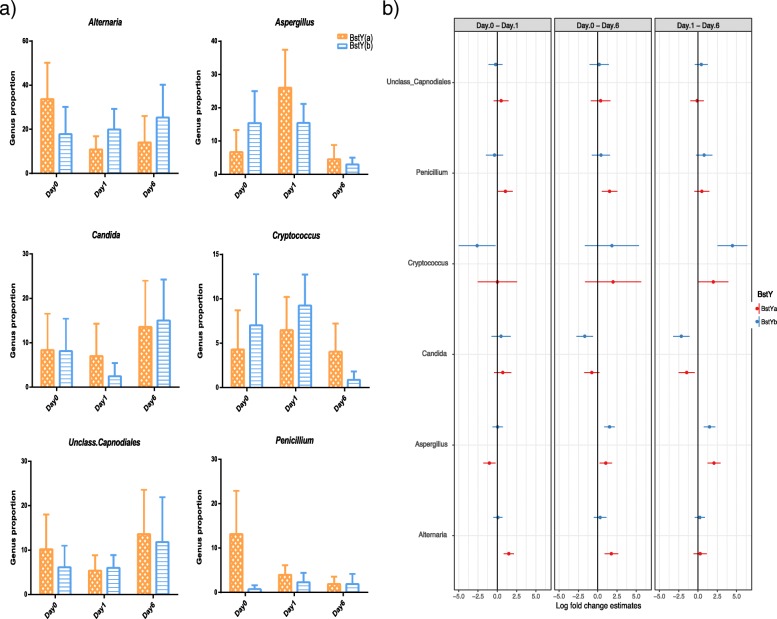
Dynamics of fungal communities of intramammary microbiota during the first week of lactation. **a** Bar graphs show the proportion of predominant fungal genera of intramammary secretions during the first week of lactation. Color codes were used to relate the average proportion of genera to BoLA variants, *x*-axis relates the proportion of bacterial genera to days in milk, and error bars denote 95% confidence intervals. **b** Associations of bacterial genera with BoLA variants and DIM were analyzed with generalized linear mixed effect model (package glmmTMB). The total count of OTUs assigned to each genus were offset to the library size (total count of OTUs detected in each sample) and then used as the response variable in a negative binomial model where BoLA variants, DIM, and their interaction were included as fixed effects, whereas the effect of individual cows were included as the random effect. Estimated group means, confidence intervals (CI), and pairwise comparisons for effects of BstY variants and DIM were derived using the package emmeans. Multiple hypotheses were adjusted by Benjamini and Hochberg false discovery rate (FDR). Genera for which the high and low values of CI do not cross the zero line show significant log fold change between DIM contrasts. Summary statistics are available in Additional file 2: Table S4

The fungal communities of colostrum and milk samples were mainly composed of phylum Ascomycota, followed by considerably lower proportions of genera belonging to phyla Basidiomycota and Zygomycota. Similar to bacterial community, dominant fungal genera of mammary secretions showed different trajectory patterns over the first week of lactation and in relation to BoLA variants (Fig. 5a, b). For example, *Alternaria*, the predominant genus within day 0 colostrum microbiota of both BstY variants, showed a decreasing pattern during the first week of lactation in the microbiota of BstYa variant, whereas the proportion of this genus remained unchanged in the microbiota of BstYb variant during the same time period. On the other hand, proportions of genera *Aspergillus* and *Cryptococcus* showed similar decreasing pattern following transition from colostrum to day 6 milk in BstYb variant. The proportion of *Candida* increased from day 1 to day 6 in the microbiota of both BoLA variants. Summary statistics for all pairwise contrasts are presented in Additional file 2: Table S4.

### Association of *BoLA*-*DRB3.2* polymorphism with the composition of day 0 colostrum microbiota

**Fig. 6:**
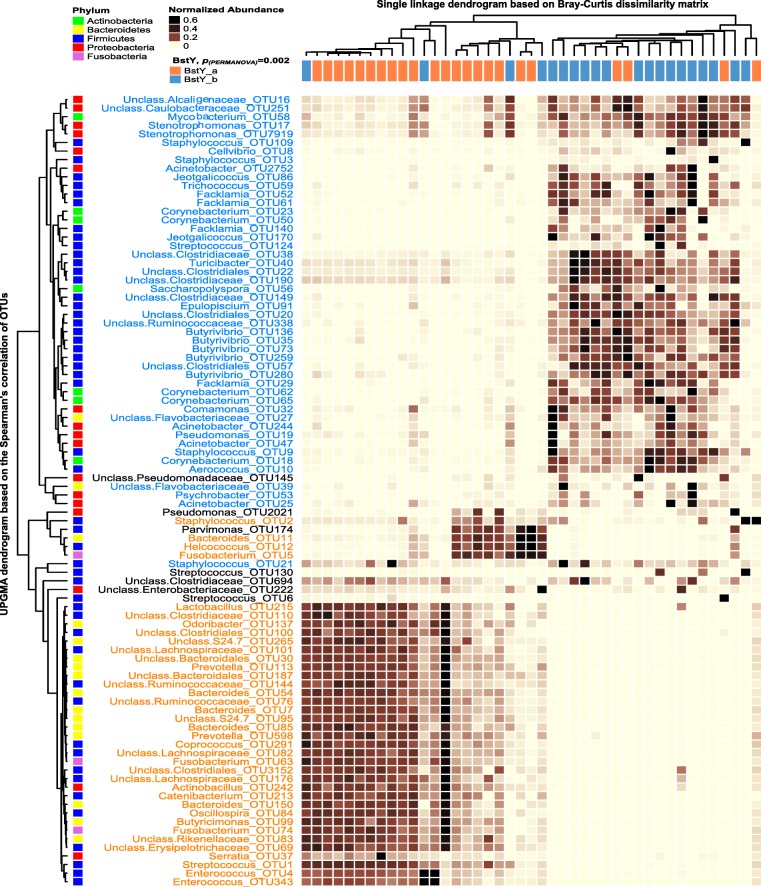
Unsupervised cluster analysis of day 0 colostrum samples based on the distribution of bacterial OTUs. Rows correspond to individual bacterial OTUs (relative abundance > 0.1% of the community). Columns correspond to individual samples, color coded based on *BoLA-DRB3.2* variants (BstYa) and (BstYb). The “Normalized Abundance” key relates colors to the normalized proportions of OTUs across samples. The top dendrogram shows the result of single-linkage hierarchal clustering of samples based on the Bray-Curtis dissimilarities of their bacterial communities. The left dendogram shows how OTUs correlate (co-occur) with each other based on their Spearman’s correlation coefficient. The “Phylum” key relates the left annotations to the corresponding phylum of each OTU. Color codes have been used to highlight statistically significant associations between the proportion of OTUs and *BoLA*-*DRB3.2* variants (identified using linear discriminant analysis effective size (LEfSe)). Bray-Curtis resemblance matrix was created based on the proportions of the abundant bacterial OTUs and subjected to PERMANOVA (9999 permutations) in order to test the significance of the clustering pattern of samples based on *BoLA*-*DRB3.2* variants

Unsupervised hierarchical cluster analysis (UPGMA) based on the proportions of most abundant bacterial OTUs (> 0.1% of the community) was performed to test whether samples belonging to the two BoLA variants tend to cluster distinctly based only on the proportion of abundant bacterial OTUs. This resulted in generation of two main clusters of samples corresponding to the two BoLA variants. In order to test the significance of clustering pattern of the two BoLA variants based on the proportion of abundant OTUs, the Bray_Curtis dissimilarity matrix of samples was calculated and subjected to PERMANOVA, resulting in identification of distinct clustering pattern (*p*_(PERMANOVA)_ = 0.002; Fig. 6). Statistical comparison (LEfSe) at the OTU level revealed that the proportions of several non-*aureus* staphylococci (NAS) OTUs, including OTU109 (*S*. *equorum*), OTU3 (*S*. *gallinarum*), OTU9 (*S*. *sciuri*), and OTU21 (*S. haemolyticus*), *Streptococcus* OTU124 (*S*. *equinus*), actinobacterial OTUs belonging to *Corynebacterium* and *Mycobacterium*, and proteobacterial OTUs belonging to udder-associated opportunistic/pathogenic bacteria such as *Stenotrophomonas*, *Pseudomonas*, *Acinetobacter*, and Alcaligenaceae were all enriched within the colostrum microbiota of day 0 colostrum samples of BstYb variant (Fig. 6; NCBI BLASTN bit-scores for species level classification are presented in Additional file 2: Table S5). On the other hand, day 0 colostrum samples of BstYa variant were enriched with *Streptococcus* OTU1 (*S*. *dysgalactiae*), *Staphylococcus* OTU2 (*S*. *chromogenes*), several *Fusobacterium* OTUs, and OTUs belonging to lactic acid bacteria (LAB) including *Lactobacillus* and *Enterococcus*. Other than abovementioned bacterial OTUs, the proportion of several gut-associated bacterial OTUs were also enriched within the colostrum microbiota of each *BoLA*-*DRB3.2* variant; Firmicutes OTUs belonging to *Butyrivibrio* and Clostridiales were enriched within the colostrum microbiota of BstYb variant, whereas Bacteroidetes OTUs belonging to *Bacteroides* and *Prevotella* were overrepresented within the microbiota of BstYa variant (Fig. 5).

UPGMA based on the proportion of abundant fungal OTUs (> 0.1% of community) revealed no distinct clustering pattern between the mycobiota of different BoLA-DRB3.2 variants (Additional file 1: Figures S6 and S7). This was further confirmed by complementary PERMANOVA test on the Bray-Curtis dissimilarity matrix of samples based on the proportion of abundant OTUs (day 0, *p*_(PERMANOVA)_ = 0.098; day 1, *p*_(PERMANOVA)_ = 0.25). Nonetheless, statistical comparison (LEfSe) revealed enrichment of several fungal OTUs within the mycobiota of each variant. On day 0, the proportions of several *Penicillium* OTUs (including OTU32: *Penicillium ilerdanum*, OTU22: *Penicillium psychrosexualis*, and OTU556: *Penicillium chrysogenum*) were enriched within the colostrum microbiota of BstYa variant (Additional file 1: Figure S6). On the other hand, on day 1, proportions of *Alternaria* OTUs (including OTU6: *Alternaria infectoria* and OTU177: Unclassified *Alternaria*) were higher within the colostrum microbiota of BstYb variant (Additional file 1: Figure S7).

### Association of *BoLA*-*DRB3.2* polymorphism with co-occurrence patterns of intramammary bacterial communities

**Fig. 7:**
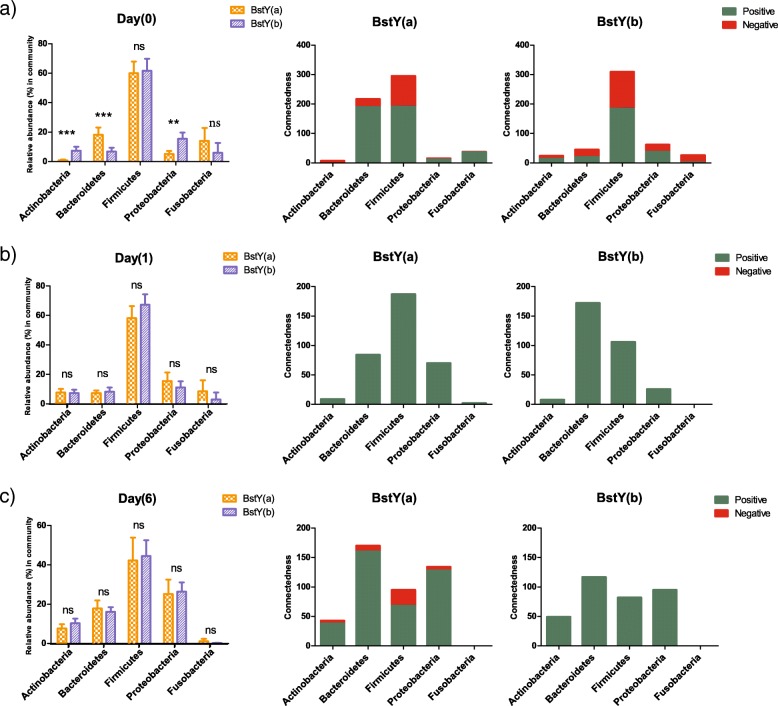
Association of *BoLA*-*DRB3.2* polymorphism with interrelationship dynamics of intramammary bacterial communities. Co-occurrence network inference (CoNet) was used to measure the impact of *BoLA*-*DRB3.2* polymorphism on the interrelationship (connectedness) patterns of intramammary bacterial communities during the first week of lactation. The grouped bar charts on the left side of each row show the average proportions of bacterial phyla within the intramammary microbiota of different *BoLA*-*DRB3.2* variants (BstYa and BstYb) at different sampling time points (**a** day 0, **b** day 1, **c** day 6). Stacked bar charts in the middle and on the right side of each row show the total number of connections (significant positive or negative relationships) observed among OTUs belonging to major bacterial phyla within the microbiota of each *BoLA*-*DRB3.2* variant. Color codes have been used to depict the type of relationships; green for positive (co-occurrence) and red for negative (mutual exclusion) relationships

Correlation network analysis was further performed to evaluate potential association of *BoLA*-*DRB3.2* polymorphism with co-occurrence patterns of intramammary bacterial communities (Fig. 7a–c, and Additional file 1: Figures S4 and S5a–f). By using an ensemble method (a combination of diverse measures of correlation, i.e., Pearson and Spearman, and dissimilarity, i.e., Bray-Curtis and Kullback-Leibler), we were able to track changes in the co-occurrence patterns of bacterial OTUs within the microbiota of each BoLA variant at different time points. Similar to the trends observed for alpha- and beta-diversity analyses, the association of *BoLA*-*DRB3.2* with the structure of microbe-microbe relationships was more pronounced within the microbiota of day 0 colostrum samples than day 1 or 6. On day 0, Firmicutes OTUs played central role in the overall structure of the bacterial community of BstYb variant, while the contributions of OTUs from other bacterial phyla were negligible. Indeed, with the exception of two OTUs belonging to *Fusobacterium* and *Bacteroides*, the entire hub OTUs (i.e., OTUs showing the highest number of positive/negative connections with other members of the community) within day 0 microbiota of BstYb variant belonged to Firmicutes, more specifically, genera *Streptococcus*, *Butyrivibrio*, *Enterococcus*, *Helcococcus*, and unclassified Clostridiaceae. On the other hand, within day 0 microbiota of BstYa variant, Firmicutes and Bacteroidetes OTUs showed equal number of connections with other members of the community. Hub OTUs within this group belonged to Firmicutes (including genera *Oscillospira*, unclassified Clostridiaceae, and unclassified Ruminococcaceae), Bacteroidetes (including genera *Bacteroides*, *Prevotella, S24*–*7*, and unclassified Bacteroidales), and *Fusobacterium*.

Following the transition from colostrum to milk, the proportions of main bacterial phyla within the microbiota of the two *BoLA*-*DRB3.2* variants became similar to each other, and so did the overall co-occurrence pattern of different bacterial OTUs belonging to these phyla (Fig. 7a–c). Although Firmicutes continued to remain the most dominant bacterial phylum within intramammary microbiota of both *BoLA*-*DRB3.2* variants, overall connectedness of Bacteroidetes and Proteobacteria OTUs took over those of the Firmicutes OTUs by progression of DIM. In addition, during this transitional period, the total number of negative connections (mutual exclusion relationships) of intramammary bacterial communities was generally reduced. On day 6, the hub OTUs within the bacterial communities of both variants became more or less similar to each other, with several *Sphingobacterium* OTUs, including OTU75 (*S*. *daejeonense*) and OTU45 (*S*. *alimentarium*) showing the highest number of connections within both communities. Nonetheless, day 6 microbiota of each variant also contained few exclusive hub OTUs, including *Staphylococcus* (OTU9) within the microbiota of BstYa variant and *Microbacterium* (OTU103) and *Acinetobacter* (OTU25) within the microbiota of BstYb variant (Additional file 1: Figure S4).

## Discussion

Although the impacts of environmental drivers, such as housing conditions and antimicrobial use, on the composition of bovine milk microbiota have been described [29, 34–36], it remains unknown whether cows’ genotype can also play a role in modulating the diversity of microbial communities of intramammary secretions. In the present study, we demonstrated that *BoLA*-*DRB3.2* polymorphism is strongly associated with the composition of both bacterial and fungal communities of intramammary secretions during the first week of lactation. Particularly, we observed that the distribution of dominant bacterial OTUs were widely different between the colostrum microbiota of the two identified *BoLA*-*DRB3.2* variants, suggesting that variations in BoLA class II molecules may result in colonization resistance against certain microbial lineages within the MG ecosystem. In addition, by characterizing the microbiota profile of intramammary secretions collected at multiple sampling time points during the first week of lactation, our study provides novel insights into the dynamics of bacterial and fungal communities of intramammary secretions during transition from colostrum to milk.

### Association between BoLA polymorphism and intramammary microbiota

The contribution of local immunity of MG in modifying intramammary microbiota is unknown. MHC polymorphism can influence the composition of mucosa-associated microbiota via inducing biases in the selective activity of secretory IgAs against bacterial surface epitopes [13, 37]. Secretory IgA is the predominant immune component of the mucus layer responsible for exclusion of exogenous antigens [38]. However, due to the very low concentrations of IgA in bovine colostrum and milk (approximately 3.5 and 0.13 mg/mL, respectively [39]), it is less likely that this antibody can serve as the main driver of BoLA-mediated shifts in the composition of mammary microbiota. Conversely, bovine colostrum contains high concentrations of IgG (32–212 mg/mL) [39]. IgG can participate in MHC-mediated selective immune responses against exogenous antigens via inactivation of bacterial antigens by binding to specific surface sites, bacterial opsonization, and complement activation [40, 41]. IgG1, the predominant subclass of IgG in bovine colostrum (~ 80% of total Igs), is mainly derived from blood, whereas IgG2, as well as IgA and IgM, are mainly synthesized by plasma cells or epithelial cells of the MG [42]. In the present work, the influence of *BoLA*-*DRB3.2* polymorphism on the composition of intramammary microbiota was more evident on day 0 colostrum samples and faded abruptly thereafter. This trend is very similar to peripartal fluctuations in the concentration of intramammary IgG1. During the prepartum period, IgG1 is gradually accumulated in colostrum in order to prepare for passive transfer of maternal immunity to newborn calves [43]; however, transition from colostrum to milk is accompanied by a rapid drop in intramammary concentrations of IgG1 [39, 44]. An important limitation of the present study was the lack of measurement of immunomodulatory components of intramammary secretions, most importantly IgGs concentrations. Nonetheless, we speculate that during the prepartum period, prolonged isolation of mammary gland from environmental sources of microbes (i.e., due to teat canal closure) provides an ideal ecosystem for IgG1 to exert BoLA-mediated shifts in the composition of persistent intramammary microbiota. Nonetheless, BoLA class II expression by epithelial cells of the MG and/or intramammary lymphocytes also has the potential to influence local immune responses and therefore modify the composition of microbiota. To what extent each of the abovementioned systemic and local immune responses contribute to regulation of intramammary microbiota remains largely unknown.

The main biologic relevance of BoLA polymorphism for udder health is modulation of mastitis susceptibility. In dairy cows, allelic variations of *BoLA*-*DRB3.2* have been associated with susceptibility to IMI by mastitis pathogens and/or severity of mastitis [20–24]. Yet, underlying mechanisms remain unknown. Recently, Kubinak et al. [13] demonstrated that MHC-mediated shifts in the composition of mucosa-associated microbiota can result in colonization resistant against *Salmonella enterica* serovar Typhimurium infection in mice. The authors suggested an indirect mechanism by which MHC gene polymorphism can modulate infection susceptibility by altering the robustness of the intestinal microbiota rather than direct involvement of the immune system. In the present study, increased proportions of LAB, especially *Lactobacillus* and *Enterococcus*, within the colostrum microbiota of BstYa variant was concomitant with decreased proportions of several important bacterial groups that are commonly regarded as mastitis pathogens or opportunists, including *Staphylococcus*, *Streptococcus*, *Mycobacterium*, and *Corynebacterium*. With the exception of few pathogenic strains, *Lactobacillus* and *Enterococcus* strains isolated from bovine milk are commonly regarded as beneficial bacteria capable to inhibit the growth of mastitis pathogens via the production of inhibitory substances such as bacteriocins, organic acids, and hydrogen peroxide [45–47]. On the other hand, corynebacterial and staphylococcal strains isolated from bovine milk, particularly NAS, are also capable to produce a wide range of antimicrobial molecules [48, 49]. Our results suggest that each of the *BoLA*-*DRB3.2* variants may promote the growth of different NAS species; *S*. *equorum*, *S. gallinarum*, *S. sciuri*, and *S*. *haemolyticus* were all enriched within the colostrum microbiota of BstYb variant, whereas *S*. *chromogenes* was enriched within the colostrum microbiota of a sub-group of cows within the BstYa variant. Altogether, these observations may be indicative of development of specific patterns of microbiota-derived colonization resistant that are mediated by *BoLA*-*DRB3.2* polymorphism. Nonetheless, it is also possible that variations in MHC class II expression directly influence the composition of microbiota by activating different profiles of pro- and anti-inflammatory responses and therefore exerting positive (tolerance) or negative (clearance) effect on different bacterial groups [10]. Given the important implications that microbiota-derived resistance against colonization by mastitis pathogens can have for development of alternative preventive therapeutics, this hypothesis warrants further mechanistic investigations of potential links among BoLA gene polymorphisms, immunomodulatory components of the immune system, and intramammary microbiota.

An important caveat of the present study was the inability to assess potential impacts of *BoLA*-*DRB3.2* polymorphism on the stability and resilience of the intramammary microbiota beyond the first week of lactation. Indeed, the main objective of the present study was to provide evidence for potential links between cows’ genotype and intramammary microbiota in the first place. Otherwise, assessment of the impact of *BoLA*-*DRB3.2* polymorphism on the resilience of intramammary microbiota would have required quarter-based longitudinal milk sampling over a relatively long period of time during early and mid stage of lactation. Notwithstanding this limitation, comparison of the interrelationship networks of the microbial communities during the first week of lactation suggests that BoLA-mediated effects on the structure of intramammary microbiota are not long-lasting. It appears that during the colostrogenesis period, when accumulation of immune components results in a high selective pressure on the microbial ecosystem of the MG, community assembly process follows a “habitat filtering” pattern in which phylogenetically related species that possess specific traits required for survival under certain environmental conditions (i.e., the selective pressure of the immune system) tends to co-occur [50]. This might be the reason why day 0 microbiota of *BoLA*-*DRB3.2* variants showed distinct clustering patterns, each orchestrated by a completely different set of hub OTUs. It is also noteworthy to mention that other than potential contribution of selective pressure (i.e., immunomodulatory components of the milk), other stochastic ecological processes, such as drift [51], might have also contributed to shaping the composition of the isolated microbial ecosystem of the mammary gland during the dry period.

### Dynamics of intramammary microbiota during transition from colostrum to milk

During the early stages of lactation, the changeover from colostrum to transitional and then mature milk is accompanied by considerable shifts not only in the concentrations of immune components [39, 44] but also other bioactive compounds such as milk oligosaccharides [52]. Many of these factors have the ability to impact the composition of mammary and intestinal microbiota [53, 54] via several mechanisms such as the ability of milk oligosaccharides to compete with microbes over attachment to epithelial cell surface receptors and therefore confer colonization resistance [55]. In addition, lactation increases the exposure of intramammary ecosystem to environmental sources of microbes including, among others, milking equipment and bedding materials. These environmental factors have also proven to be influential in modifying the composition of intramammary microbiota [34, 56]. Prior to entering the sampling procedure of the present study, selected cows were subjected to blanket dry cow therapy using a combination of internal teat sealant and long-lasting antimicrobial. Not surprisingly, we observed that transition from colostrum to milk, which coincided with the exposure of isolated mammary gland to environmental sources of microbes (i.e., milking equipment, bedding material, etc.), resulted in significant increases in the diversity of both bacterial and fungal components of the intramammary microbiota. Regarding the bacterial communities of colostrum microbiota, our results are in partial agreement with the observations of Lima et al. [30] who also reported the predominance of *Staphylococcus* and gut-associated bacteria including Ruminococcaceae and Clostridiales in the colostrum microbiota of both primiparous and multiparous cows. *Staphylococcus* spp., in particular NAS, are predominant colonizer of bovine MG commonly considered as the main causes of subclinical mastitis [57]. In the present study, we observed that *Staphylococcus* was the most abundant bacterial genera throughout the first week of lactation within the microbiota of both BoLA variants. *Fusobacterium* was another dominant member of the colostrum microbiota the proportion of which remained unchanged during the first week of lactation. Lima et al. [30] reported that the colostrum microbiota of MG quarters that had mastitis during the early stages of lactation contained a higher proportion of *Fusobacterium* and were significantly less diverse than the microbiota of quarters that did not experience clinical mastitis. In our study, the colostrum microbiota of the BstYa variant, which contained a higher proportion of phylum Fusobacteria, was significantly less diverse than BstYb variant. However, the composite milk sampling procedure of our study precluded us form relating this observation with future incidences of clinical mastitis. Another notable dynamic in the bacterial communities during transition from colostrum to milk was enrichment of the genus *Sphingobacterium*. Indeed, our correlation network analysis revealed that on day 6, OTUs belonging to this genus were among the most influential (hub) OTUs in shaping the overall structure of the milk microbiota. This is in agreement with the result of our parallel study [58], where we observed positive associations between the proportions of hub Sphingobacterial OTUs and teat-end hyperkeratosis or future incidences of clinical mastitis. Oikonomou et al. [28] also reported a strong correlation between the proportion of *Sphingobacterium* and increased milk somatic cell count. Others [59, 60] have also detected members of this bacterial genus in the milk samples obtained from clinical and subclinical cases of mastitis. All together, these observations suggest that *Sphingobacterium* is a potent colonizer of the MG and perhaps an emerging mastitis pathogen in different countries.

Our study provides the first report on the composition of fungal microbiota of intramammary secretions. Fungi are ubiquitously present in dairy environment and therefore cross-contamination of samples with environmental fungi through air-born spores and/or aerosols during sampling might be inevitable under field conditions. Nonetheless, our results indicated significant impact of *BoLA*-*DRB3.2* polymorphism on the composition of fungal component of colostrum microbiota. This observation suggests the existence of a resident, perhaps commensal, community of fungi within the MG ecosystem. The prevalence of mycotic mastitis is relatively low (~ 7–12% [61, 62]); however, strains belonging to genera *Candida*, *Cryptococcus*, *Alternaria*, and *Aspergillus* have been commonly isolated from bovine milk [62–65]. Here, we also observed that *Alternaria*, *Aspergillus*, *Candida*, and *Cryptococcus* were the most abundant fungal components of intramammary microbiota during the first week of lactation. Compared to the bacterial community, the composition of fungal community was overall more stable during transition from colostrum to milk. *Scedosporium* was the only abundant fungal genera the proportion of which increased during this transitional phase. *Scedosporium* spp. have been characterized as opportunistic pathogens with the ability to cause infection in immunocompromised animals and humans [66, 67]. Our observation suggests that this genus might be an environmental opportunistic that is capable to colonize the MG after the dry period and following exposure of the intramammary ecosystem to the environmental sources.

## Conclusion

The present study provides novel insights into the influence of *BoLA*-*DRB3.2* polymorphism on the composition of both bacterial and fungal components of intramammary secretions. Overall, our results indicated that BoLA-mediated shifts were more pronounced on the microbiota composition of colostrum samples rather than milk. Particularly, we observed that within colostrum microbiota of each *BoLA*-*DRB3.2* variant, specific groups of bacteria that are known to exert colonization resistance via production of antimicrobial peptides were enriched. Determining whether BoLA-mediated shifts in the composition of intramammary microbiota are regulated directly by immune system or indirectly by microbiota-derived colonization resistance can have important implications for future development of preventive/therapeutic strategies for controlling mastitis. Even if this modulatory impact is restricted to the colostrogenesis period, it can still have great implications for udder health and mastitis susceptibility as a high incidence rate of peripartal mastitis remains a major problem for dairy industry worldwide.

## Methods

### Selection of animals and sample collection

This study was conducted in a commercial 500-head milking dairy farm located in Manitoba, Canada, from December 2014 to February 2015. Late pregnant multiparous cows were identified during their last 3 weeks of pregnancy and deemed eligible for gradual enrollment in the sampling procedure of the present study based on the following criteria: presence of four quarters with healthy appearance (no visible sign of clinical mastitis such as swelling or redness, and devoid of anatomically damaged teat ends), and no incidence of clinical mastitis during the last 90 days of the previous lactation. At the end of the previous lactation cycle, all cows were subjected to blanket dry cow therapy using internal teat sealant (Orbeseal, Zoetis, CA, USA) in combination with long-acting antimicrobial product containing 200,000 units penicillin G and 400 mg novobiocin per 10 mL tube (NovaDry; Pfizer Animal Health, QC, Canada). During the late-pregnancy period till 3 days post-calving, cows were housed in designated transition pens, bedded with fresh and dry straw. Afterwards, all cows were transferred to free-stall pens bedded every other day with recycled bedding material. Following parturition, cows were milked three times a day at 4 am, noon, and 8 pm. Cows selected for the purpose of the present study (*n* = 60) were subjected to three sampling time points during the first week of lactation:( a) fresh colostrum collected within 6 h of calving (day 0), (b) colostrum collected at noon of the first day post-calving (day 1), and (c) fresh milk collected at noon of the sixth day post-calving (day 6). On the day of sampling, all four quarters of the MG of each cow were checked for clinical signs of mastitis (swelling or redness). Prior to sampling, four streams of foremilk from all quarters were discarded to (a) minimize chances of sample contamination from bacteria colonizing the teat canal, and (b) to check for abnormal appearance of colostrum/milk (i.e., watery secretions, presence of blood, flake, or abnormal color). Cows diagnosed with clinical mastitis at any sampling time point were excluded from the study (*n* = 6). For the remaining cows (*n* = 54), pre-milking teat disinfection was performed using 0.5% iodine pre-dip solution, teats were thoroughly dried using individual paper towels and then scrubbed for 15 s using cotton pads moistened in 70% alcohol. Composite colostrum and milk samples (~ 10 mL from each quarter combined into a 50 mL sterile containers) were then collected and placed on ice in the milking parlor, temporary stored at − 20 °C on the farm, and transferred over dry-ice to the laboratory for further processing.

### DNA extraction from colostrum and milk samples

Colostrum and milk samples collected from 54 multiparous cows were subjected to genomic DNA extraction using the ZR-96 Fungal/Bacterial DNA Kit (Zymo Research, Irvine, CA, USA) following modified protocols of the manufacturer as follows: 1.5 mL of each sample was centrifuged at 12,000×*g* for 20 min at 4 °C. Supernatants were carefully removed and 200 μL of TE buffer and 300 μL of 0.5 M EDTA (pH = 8) were added to the pellet. The mixture was incubated for 20 min at room temperature and again centrifuged at 12,000×*g* for 10 min. Supernatants were removed and pellets were resuspended by adding 200 μL of PBS buffer and vortexing for 30 s. Next, 1 g of autoclaved 0.5 mm silica beads, 400 μL of Lysis Solution (Zymo Research, CA, USA), and 18 μL of 20 mg/mL Proteinase K (Zymo Research, CA, USA) were added to each tube and incubated in a heated shaker at 45 °C for 45 min. Tubes were then vortexed for 2 min using a 2010 GenoGrinder (SPEX SamplePrep, NJ, USA) at 1700 stroke per minutes. Then, 400 μL of the resulting mixture was then transferred to the deep-well plate of the Fungal/Bacterial DNA Kit and extraction process continued following manufacturer protocol. Negative controls (*n* = 3) were included in the extraction process by replacing 1 mL of sterile water instead of colostrum/milk samples.

### PCR-RFLP identification of the *BoLA*-*DRB3.2* variants

Genomic variations within the exon 2 of the *BoLA*-*DRB3* were determined according to the hemi-nested PCR-RFLP method described by van Eijk et al. [68] with minor modifications. For the first round of amplification, PCR reactions contained 3.0 μL of extracted DNA from day 0 colostrum samples, 1.0 μL (5 μM) of each of the primers HLO30 (5′-ATC CTC TCT CTG CAG CAC ATT TCC-3′) and HLO31 (5′-TTT AAA TTC GCG CTC ACC TCG CCG CT-3′), 0.4 μL (20 mg/mL) BSA (ThermoFisher Scientific, Mississauga, ON, Canada), 9.6 μL nuclease-free water (ThermoFisher Scientific), and 10 μL of 5 Prime Hot MasterMix (5 Prime Inc., Gaithersburg, MD, USA). PCR conditions were set up as follows: an initial denaturing step at 94 °C for 3 min followed by ten amplification cycles at 94 °C for 25 s, 60 °C for 30 s, and 72 °C for 30 s with a final extension step at 72 °C for 5 min in an Eppendorf Mastercycler pro (Eppendorf, Hamburg, Germany). A second hemi-nested amplification round was performed using 3.0 μL of the PCR product of the first round as input genomic material. Reaction ingredients were similar to the first round except that primer HLO32 (5′-TCG CCG CTG CAC AGT GAA ACT CTC-3′) was substituted for HLO31. Reaction conditions consisted of 25 amplification cycles at 94 °C for 40 s and 65 °C for 30 s with a single final extension of 72 °C for 5 min. Further, 10 μL of the PCR products of the second amplification round were incubated with 5 units of BstYI restriction enzyme (New England Biolabs Inc., MA, USA) at 60 °C for 70 min. After incubation, reactions were incubated at 85 °C for 5 min to denature the enzyme. DNA fragments were visually detected by conducting vertical electrophoresis on Mini-Protein III unit (Bio-Rad Laboratories, Mississauga, ON, Canada) using 10% Mini-PROTEAN TBE gels (Bio-Rad Laboratories) at 300 mA for 45 min.

### PCR amplification and construction of sequencing libraries

Samples were subjected to two separate PCR rounds targeted to amplify the V1-V2 regions of the bacterial 16S rRNA genes and the internal transcribed spacer 2 (ITS2) of the eukaryotic ribosomal genes (the sequence of primers used for PCR amplification and sequencing reactions can be found in Additional file 2: Tables S6 and S7). The forward PCR primers of the V1-V2 primer set and the reverse PCR primer of the ITS2 primer set were indexed with 12-base Golay barcodes, allowing for multiplexing of samples. For each sample, the PCR reaction targeting each region was performed in duplicate and contained 3.0 μL of extracted metagenomic DNA, 1.0 μL of each forward and reverse primer (5 μM), 0.4 μL of 20 mg/mL BSA (ThermoFisher Scientific), 11.6 μL nuclease-free water (ThermoFisher Scientific), and 10 μL of 5 Prime Hot MasterMix (5 Prime Inc.). A total of three negative controls were also included in PCR reactions by replacing metagenomic DNA with 3 μL of nuclease-free water. The PCR conditions for the V1-V2 primer set consisted of an initial denaturing step at 94 °C for 3 min followed by 35 amplification cycles at 94 °C for 30 s, 55 °C for 20 s, and 72 °C for 20 s, with a final extension step at 72 °C for 5 min in an Eppendorf Mastercycler pro (Eppendorf, Hamburg, Germany). The PCR conditions for the ITS2 primer set were identical to those used for V1-V2 primer set with the exception of annealing temperature being set at 52 °C for 30 s. The sequencing libraries were then generated as explained by Derakhshani et al. [69] and sequenced using MiSeq Reagent Kits V3 (600-cycle; Illumina, San Diego, CA, USA) at the Gut Microbiome Laboratory at the University of Manitoba.

### Bioinformatics and statistical analyses

The default settings of FLASH assembler ver. 1.2.11 [70] were used to merge the overlapping paired-end Illumina fastq files. UPARSE algorithm (version 8.0.1623) [71] was used for (a) quality filtering of the reads based on maximum expected error value = 1.0, (b) identification of unique sequences, (c) abundance sorting and removal of singletons, (d) clustering the reads into operational taxonomic units (OTUs) based on 97% identity threshold, (e) de novo and reference-based chimera checking (against GOLD database [20] for V1-V2 sequences and UNITE database for ITS2 sequences [72]), and (f) construction of OTU table. For V1-V2 sequences, taxonomies were assigned using the QIIME [73] implementation of the UCLUST consensus taxonomy assigner (version = 1.2.22) [74] against the Greengenes database (release May 2013). Due to the importance of staphylococci and streptococci species to udder health and pathogenesis of mastitis, sequences of representative OTUs within these two genera were further assigned taxonomy at the species level based on top BLASTN bit-scores with minimum sequence identities of 98% to representative sequences deposited in the NCBI-16S rRNA gene database. For ITS2 sequences, taxonomies were assigned using the SortMeRNA classifier (version 2.0, 29/11/2014) [75] against the UNITE database (dynamic version 7.0) [72].

Prior to performing downstream analyses, the resulting bacterial and fungal OTU tables were filtered to remove all the samples with low sequencing depths (< 5000 sequences per sample). Community richness (Chao1) and diversity (Shannon) indices were then calculated using QIIME default scripts at even depths of 6000 and 5000 sequences per sample for bacterial and fungal OTU tables, respectively (see Additional file 1: Figure S8: rarefaction curves of richness and diversity indices of bacterial and fungal communities). Bray-Curtis dissimilarities of microbial communities were calculated following normalization of the final OTU tables using the cumulative sum scaling (CSS) transformation [76]. Jaccard binary distances of microbial communities were calculated using rarefied OTU tables at even depths of 6000 and 5000 sequences per sample for bacterial and fungal OTU tables, respectively. Normalization of the final OTU tables using principal coordinate analysis (PCoA) was applied on the resulting distance matrices to generate two-dimensional plots using default settings of the PRIMER-E software ver. 6.1.18 [77].

Unsupervised clustering analysis was performed to relate clustering patterns of samples to the proportion of the most abundant OTUs. The relative abundances of selected OTUs (> 0.1% of the community) were normalized across samples (values divided by the Euclidean length of the row vector). Bray-Curtis dissimilarities were calculated using R “vegan” package [78] and the resulting matrix was subjected to unsupervised agglomerative hierarchical clustering method (unweighted pair group method with arithmetic mean (UPGMA)) using R “dendextend” package [79] and visualized over a heatmap of the abundance matrix using R “complexheatmap” package [80].

The UNIVARIATE procedure of SAS (SAS 9.3, 2012) was used for testing the normality of residuals for α-diversity measurements. Non-normally distributed data were Box-Cox power transformed and then fitted in a repeated measurement model using MIXED procedure of SAS with DIM, BstYI variants, and the interaction between DIM and BstYI variants being treated as fixed factors and the effect of individual cows treated as the random effect nested within the BstYI variants. Permutational multivariate analysis of variance (PERMANOVA [81]; implemented in Primer6 software) was used to test for significant differences in the β-diversity of microbial communities belonging to different DIM (*n* = 3) and BstYI variants (*n* = 2). Label permutations (*n* = 9999) were used in PERMANOVA to estimate the distribution of test statistics under the null hypothesis that within-group Bray-Curtis and binary Jaccard distances were not significantly different from between-group ones. A repeated measurement PERMANOVA model was defined in which DIM, BstYI variants, and the interaction between DIM and BstYI variants were treated as fixed factors while the effect of individual cows was treated as a random factor nested within the BstYI variants. Associations of bacterial genera with BoLA variants and DIM were analyzed with a generalized linear mixed effect model (repeated measurement) using package glmmTMB [82]. The total count of OTUs assigned to each genus were offset to the library size (total count of OTUs detected in each sample) and then used as the response variable in a negative binomial model where BoLA variants, DIM, and their interaction were included as fixed effects, whereas the effect of individual cows were included as the random effect. Estimated group means, confidence intervals, and pairwise comparisons for effects of BstY variants and DIM were derived using the package emmeans [83] and visualized using the package ggplot2 [84]. Multiple hypotheses were adjusted by Benjamini and Hochberg false discovery rate (FDR [85]). Statistical comparisons between the relative abundances of bacterial/fungal OTUs and BstYI variants at selected time points were performed using linear discriminant analysis effective size (LEfSe; Segata et al. [86]). In this approach, the nonparametric factorial Kruskal-Wallis sum rank test was used to test whether the relative abundances of OTUs were differentially distributed between different classes (variants) of BstYI. This was followed by linear discriminant analysis (LDA) to estimate the effect size of each differentially abundant OTU. The threshold on the logarithmic LDA score was set at 2.0, so that only OTUs with at least 2.0 log fold change were considered to be differentially distributed between classes.

Finally, correlation network analysis (CoNet, [87]) was used to explore microbial co-occurrence/mutual exclusion relationships and identify hub OTUs that show the highest number of positive/negative correlations with other OTUs. In this ensemble method, combinations of different correlation (Pearson’s and Spearman’s rank correlation coefficient) and dissimilarity (Bray-Curtis and Kullback-Leibler) measures were used to infer co-occurrence networks. In brief, for each measure, distributions of all pairwise scores between the nodes (a node representing the relative abundance of a non-singleton OTU that was found in at least 20% of the samples) were computed. For each measure and edge (an edge representing a positive or negative correlation between two nodes), 1000 permutation was conducted (this included a renormalization step for Pearson and Spearman measures in order to address the issue of compositionality introduced by different sequencing depths for each sample). For all correlation and dissimilarity measures, *p* values were computed as the probability of the null value (represented by the mean of the null distribution) under a Gauss curve generated from the mean and standard deviation of the bootstrap distribution. Measure-specific *p* values were then merged using Brown’s method [88] and subjected to Benjamini-Hochberg’s FDR correction. An edge was considered significant and kept in the final network if (a) it was supported by at least three measures, (b) it had a merged *p* value below 0.05, and (c) it was within the 95% confidence interval defined by the bootstrap distribution.

## Additional files

Additional file 1: **Figure S1.** PCR-RFLP Identification of *BoLA-DRB3.2* variants. **Figure S2.** Beta-diversity of the bacterial communities of intramammary secretions during the first week of lactation. **Figure S3.** Beta-diversity of the fungal communities of intramammary secretions during the first week of lactation. **Figure S4.** Association of lactation stage and *BoLA-DRB3.2* polymorphism with interrelationship patterns and hub species of intramammary bacterial communities. **Figure S5.** Bacterial co-occurrence and co-exclusion networks. **Figure S6.** Unsupervised cluster analysis of day 0 colostrum samples based on the distribution of fungal OTUs. **Figure S7.** Unsupervised cluster analysis of day 1 colostrum samples based on the distribution of fungal OTUs. **Figure S8.** Rarefaction curves of richness (Observed-OTUs and Chao1) and diversity (Shannon) indices for bacterial (a-c) and fungal (d-f) communities. (PDF 6512 kb)

Additional file 2: **Table S1.** Summary statistics for comparison of alpha-diversity of microbial communities. **Table S2.** Summary statistics for comparison of beta-diversity of microbial communities (PERMANOVA test statistics). **Table S3.** Summary statistics for repeated measurement analysis of bacterial communities. **Table S4.** Summary statistics for repeated measurement analysis of fungal communities. **Table S5.** NCBI blastn search result for selected bacterial OTUs. **Table S6.** Primer design and equences for barcoded PCR amplification of the V1-V2 regions of bacterial 16S rRNA genes. **Table S7.** Primer design and sequences for barcoded PCR amplification of the ITS2 of the eukaryotic ribosomal genes. **Table S8.** Metadata used for processing of 16S rRNA gene raw sequences, bioinformatics and statistical analyses of bacterial communities. **Table S9.** Metadata used for processing of ITS2 raw sequences, bioinformatics and statistical analyses of fungal communities. (XLSX 48 kb)
