# Supplementary material for: Association of bovine major histocompatibility complex (BoLA) gene polymorphism with colostrum and milk microbiota of dairy cows during the first week of lactation
Source: Microbiome. 2018 Nov 12;6:203. doi: 10.1186/s40168-018-0586-1 (PMC6233267; doi:10.1186/s40168-018-0586-1)
Supplement: Supplementary file 1 — Figure S1. PCR-RFLP Identification of BoLA-DRB3.2 variants. Figure S2. Beta-diversity of the bacterial communities of intramammary secretions during the first week of lactation. Figure S3. Beta-diversity of the fungal communities of intramammary secretions during the first week of lactation. Figure S4. Association of lactation stage and BoLA-DRB3.2 polymorphism with interrelationship patterns and hub species of intramammary bacterial communities. Figure S5. Bacterial co-occurrence and co-exclusion networks. Figure S6. Unsupervised cluster analysis of day 0 colostrum samples based on the distribution of fungal OTUs. Figure S7. Unsupervised cluster analysis of day 1 colostrum samples based on the distribution of fungal OTUs. Figure S8. Rarefaction curves of richness (Observed-OTUs and Chao1) and diversity (Shannon) indices for bacterial (a-c) and fungal (d-f) communities. (PDF 6512 kb) [file 40168_2018_586_MOESM1_ESM.pdf]

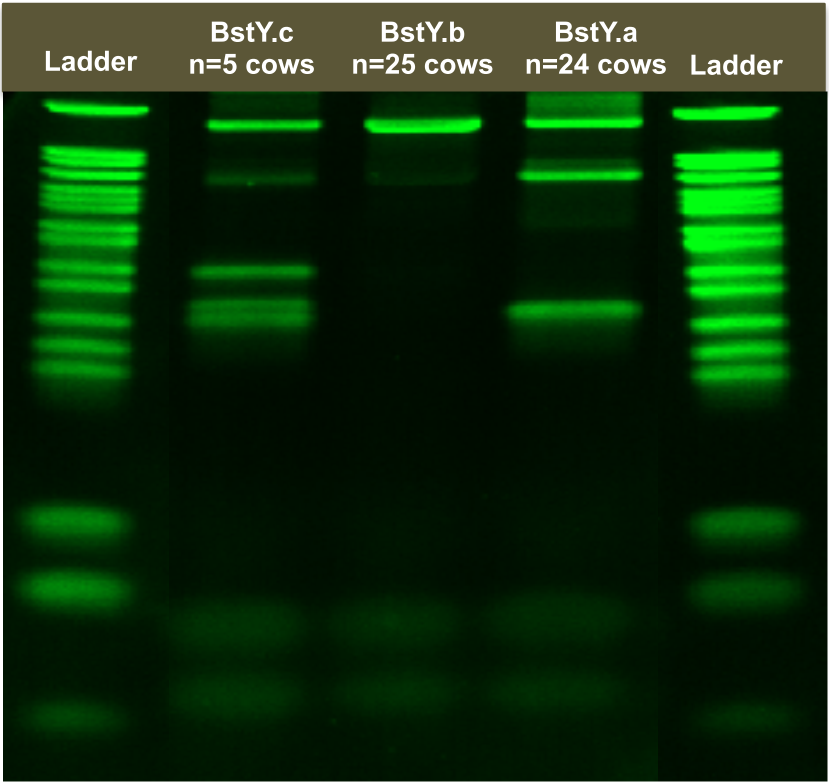

Fig S1. Identification of *BoLA-DRB3.2* variants*.* Based on the digestion activity of the BstYI enzyme (New England Biolabs Inc., MA, USA), three distinct *BoLA-DRB3.2* variants were identified within the studied population of cows (Additional File 4), named BstYa (n = 24), BstYb (n = 25), and BstYc (n = 5). DNA fragments were visually detected by conducting vertical electrophoresis on Mini-Protein III unit (Bio-Rad Laboratories, Missisauga, ON, Canada) using 10% Mini-PROTEAN® TBE gels (Bio-Rad Laboratories) at 300 mA for 45 min. pBR322 DNA-MspI Digest (New England Biolabs Inc., MA, USA) was used as ladder for measurement of DNA fragment size.

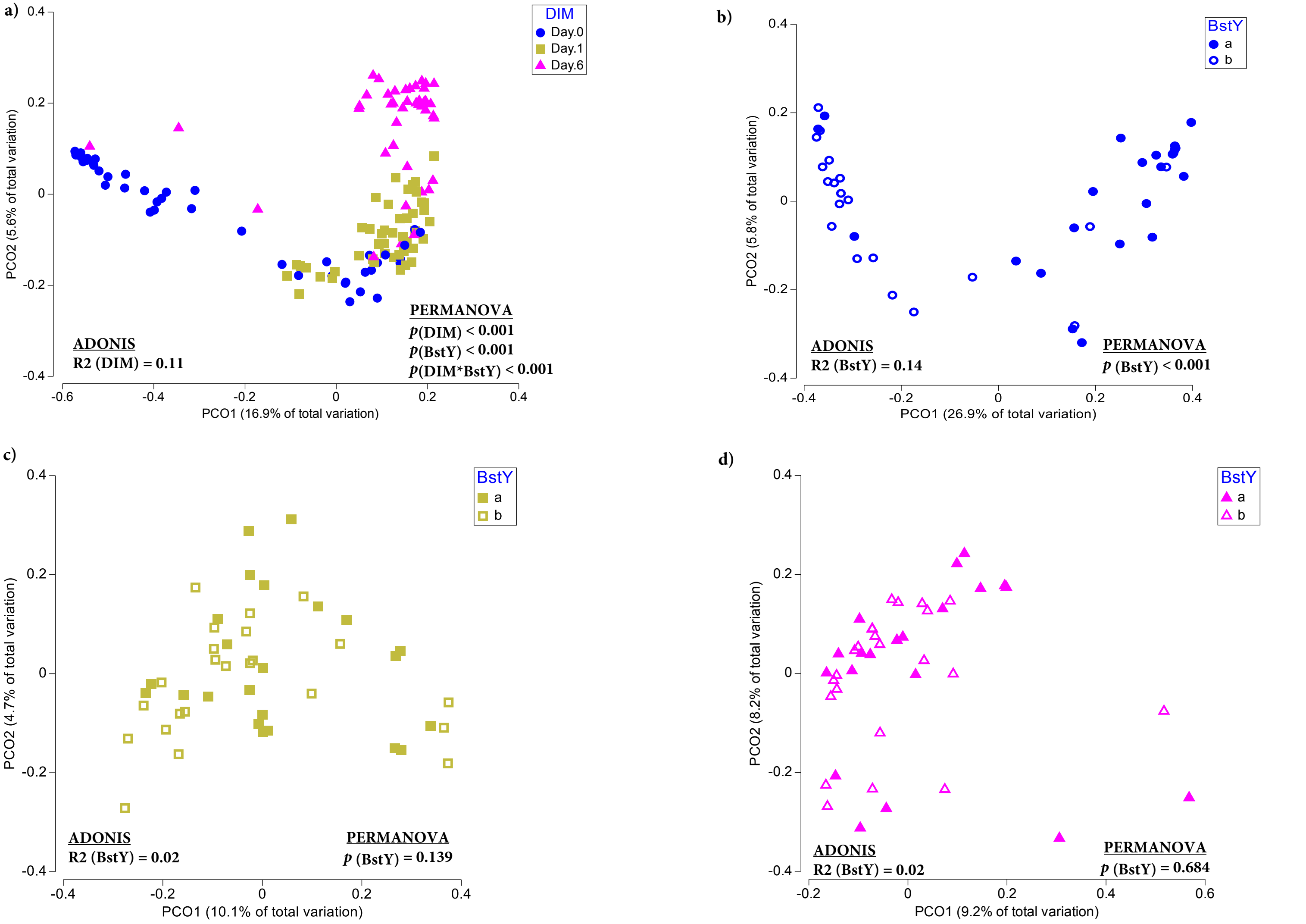

Fig. S2. Beta-diversity of the bacterial communities of intramammary secretions during the first week of lactation. Principal coordinate analysis (PCoA) was used for visualization of Jaccard binary distances of the bacterial communities of intramammary secretions. Color codes and symbols were used to differentiate samples based on a) days in milk (DIM), b) BstYI variants within day 0, c) BstYI variants within day 1, and d) BstYI variants within day 6. Prior to calculation of Jaccard distance matrix, the OTU table was rarefied at an even depth of 6000 sequences/sample. PERMANOVA (9999 permutations) was performed on a repeated measurement model that included DIM, BstYI variants, and the interaction between DIM and BstYI variants as fixed factors and the effect of individual cows as a random factor nested within the BstYI variants. For all tests, *p*-values < 0.05 were considered significant.

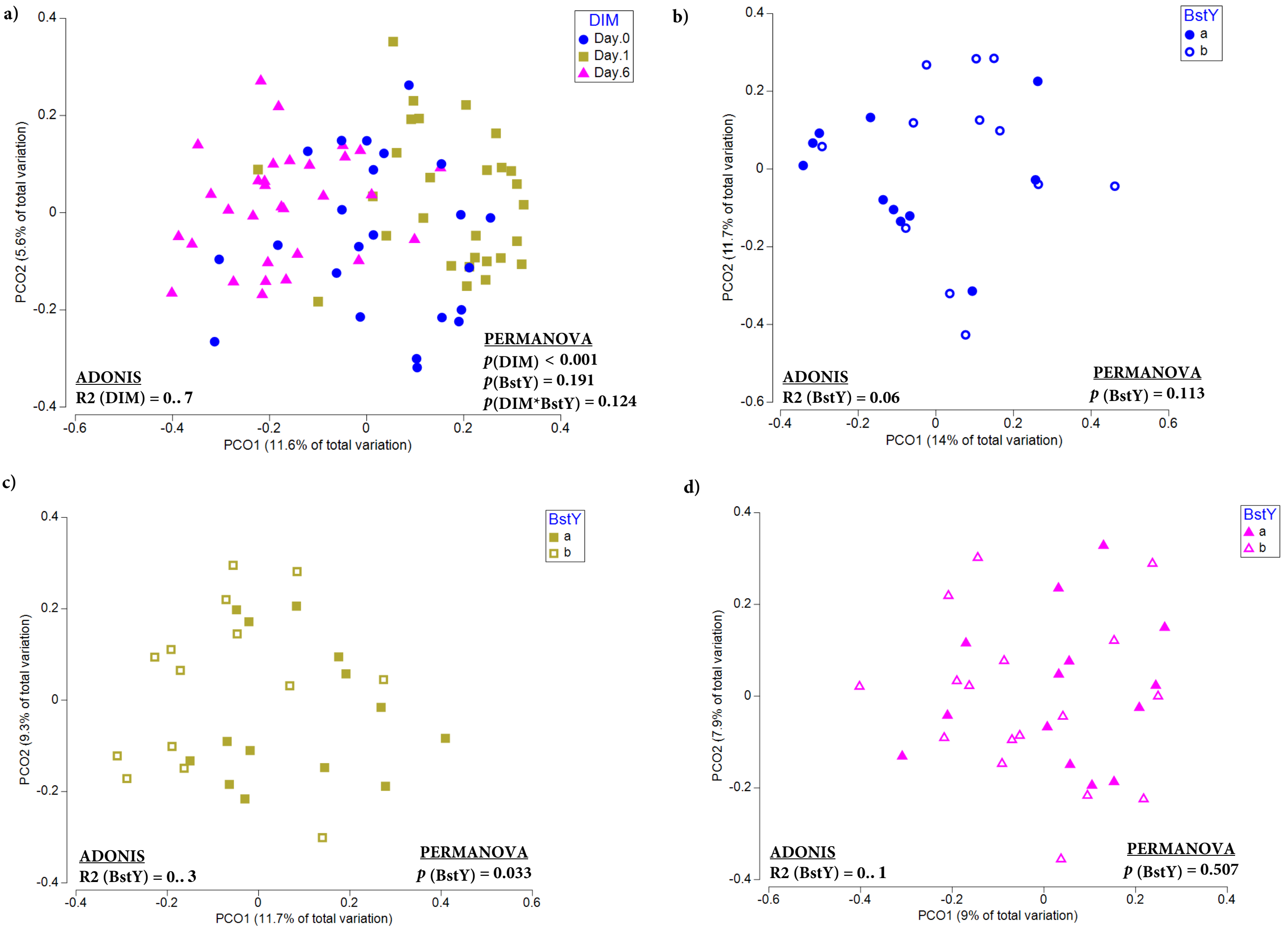

Fig. S3. Beta-diversity of the fungal communities of intramammary secretions during the first week of lactation. Principal coordinate analysis (PCoA) was used for visualization of Jaccard binary distances of the fungal communities of intramammary secretions. Color codes and symbols were used to differentiate samples based on a) days in milk (DIM), b) BstYI variants within day 0, c) BstYI variants within day 1, and d) BstYI variants within day 6. Prior to calculation of Jaccard distance matrix, the OTU table was rarefied at an even depth of 5000 sequences/sample. PERMANOVA (9999 permutations) was performed on a repeated measurement model that included DIM, BstYI variants, and the interaction between DIM and BstYI variants as fixed factors and the effect of individual cows as a random factor nested within the BstYI variants. For all tests, *p*-values < 0.05 were considered significant.

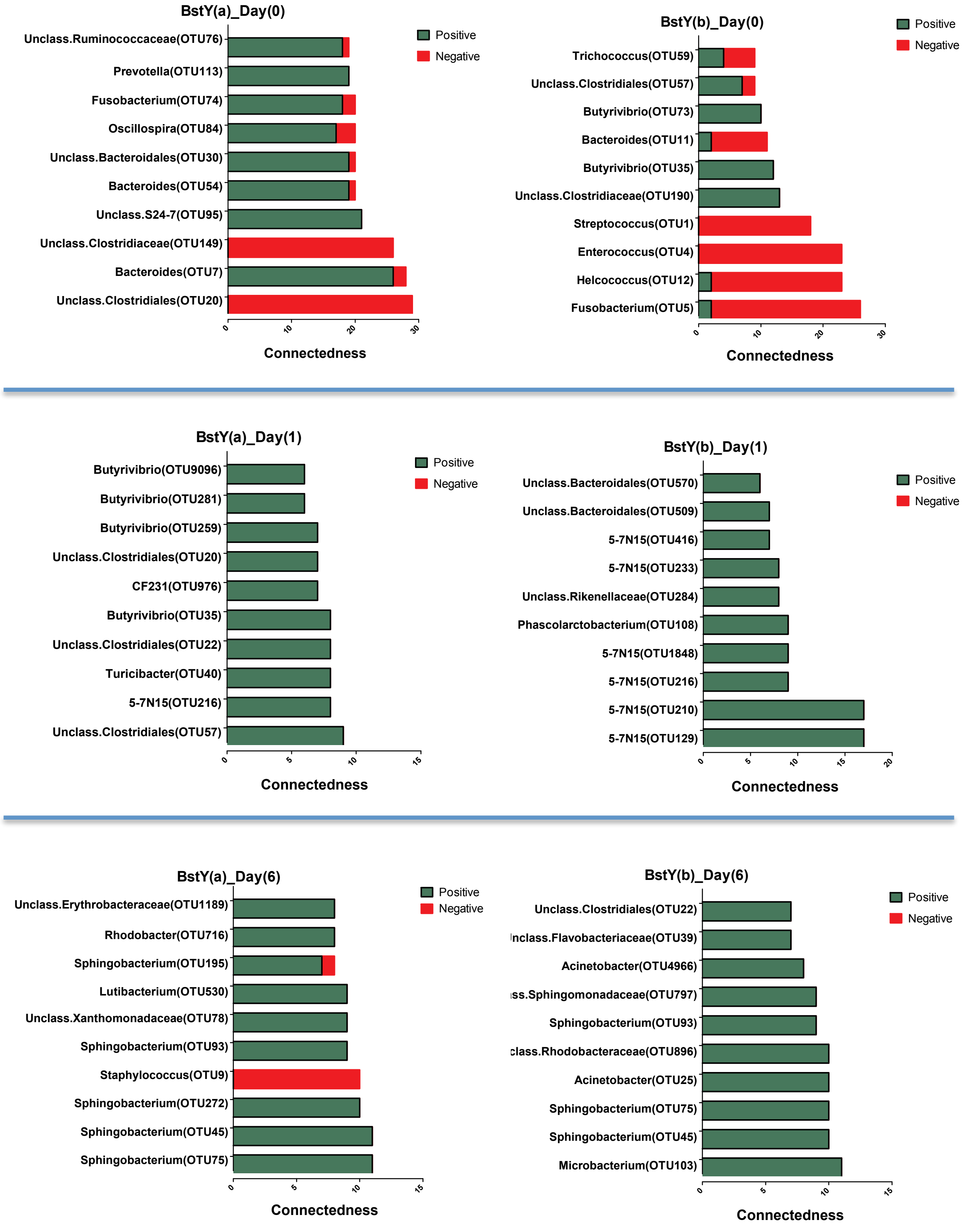

Fig S4. Association of lactationstage and *BoLA-DRB3.2* polymorphism with interrelationship patterns and hub species of intramammary bacterial communities. Co-occurrence Network inference (CoNet) was used to measure the impact of *BoLA-DRB3.2* polymorphism on the interrelationship (connectedness) patterns of intramammary bacterial communities during the first week of lactation. Stacked bar charts show the hub bacterial OTUs (i.e. those showing the highest number of significant positive or negative relationships) within the microbiota of each *BoLA-DRB3.2* variant at different sampling time points (a-c: days in milk 0, 1, and 6). Color codes have been used to depict the type of relationships; green for positive (co-occurrence) and red for negative (mutual exclusion) relationships.

a) Day 0 intramammary microbial network for BstY(a)

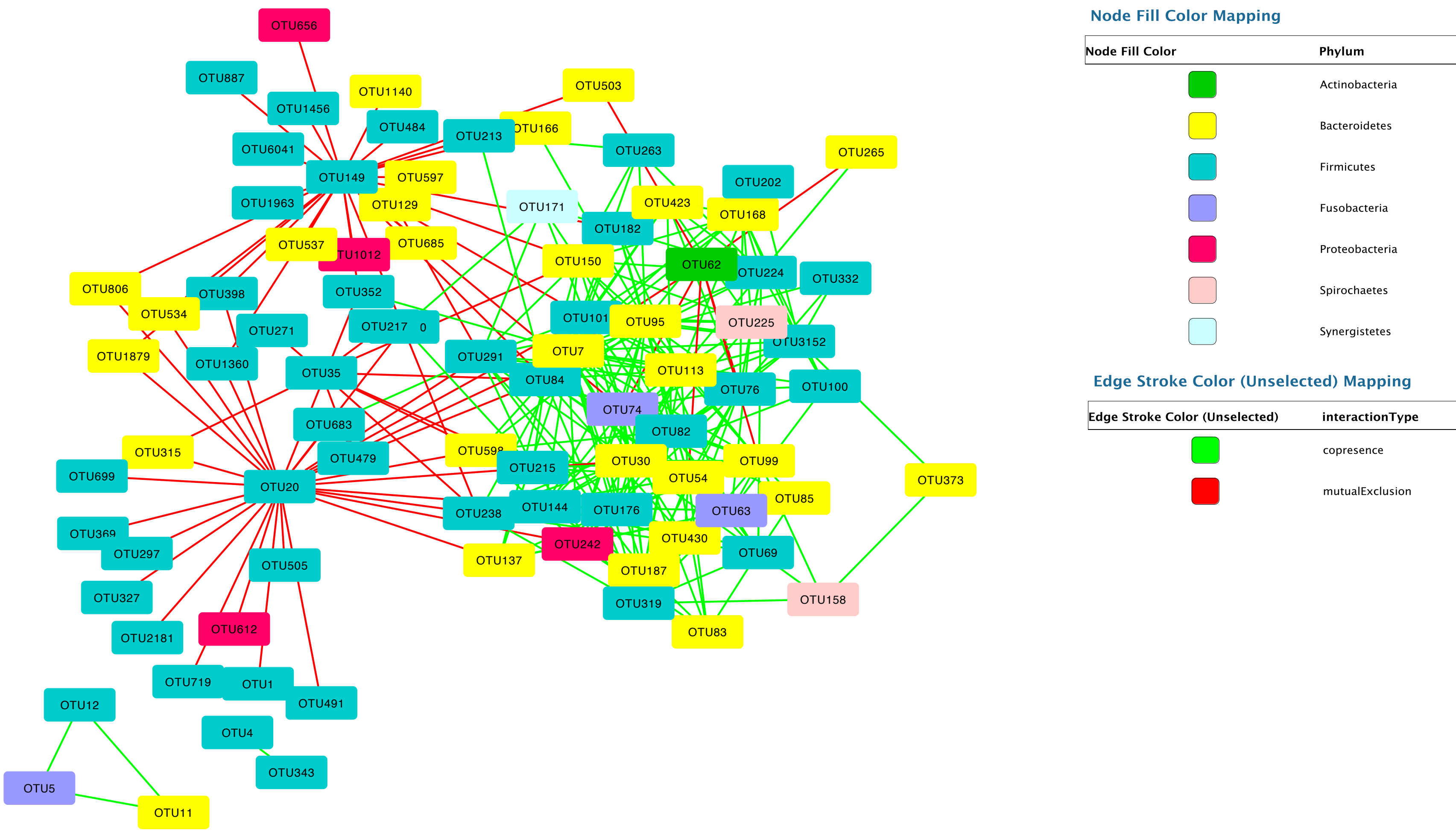

b) Day 0 intramammary microbial network for BstY(b)

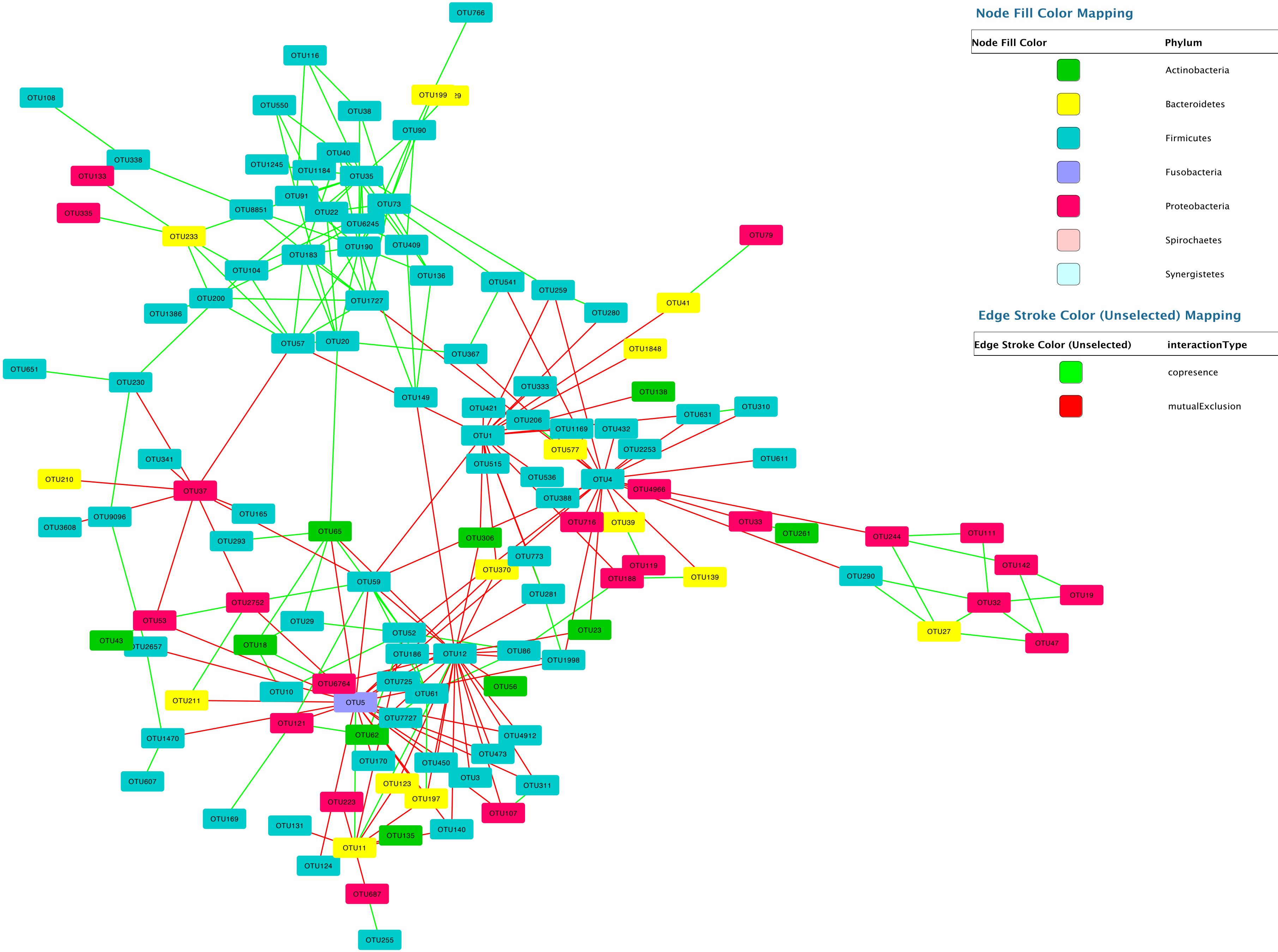

c) Day 1 intramammary microbial network for BstY(a)

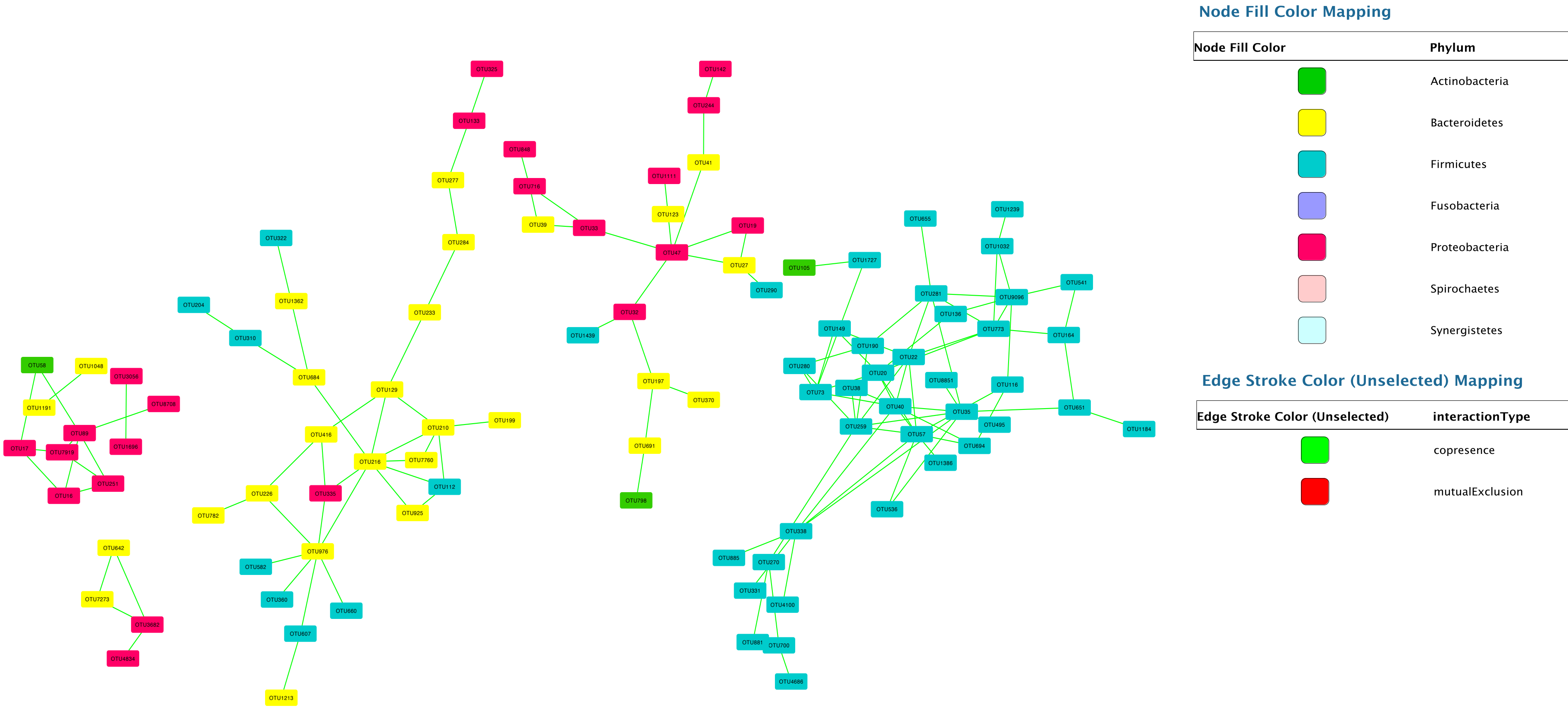

d) Day 1 intramammary microbial network for BstY(b)

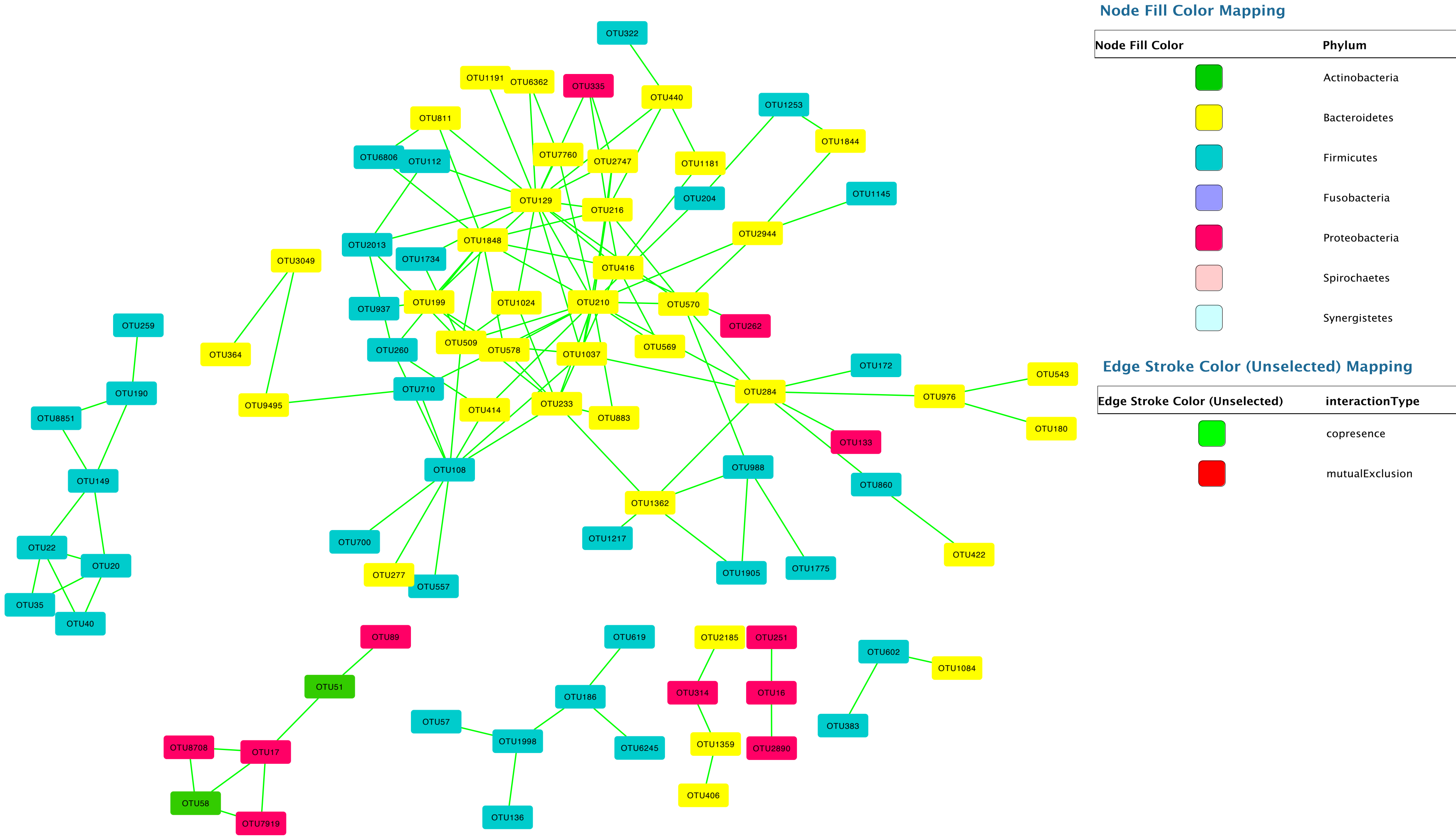

e) Day 6 intramammary microbial network for BstY(a)

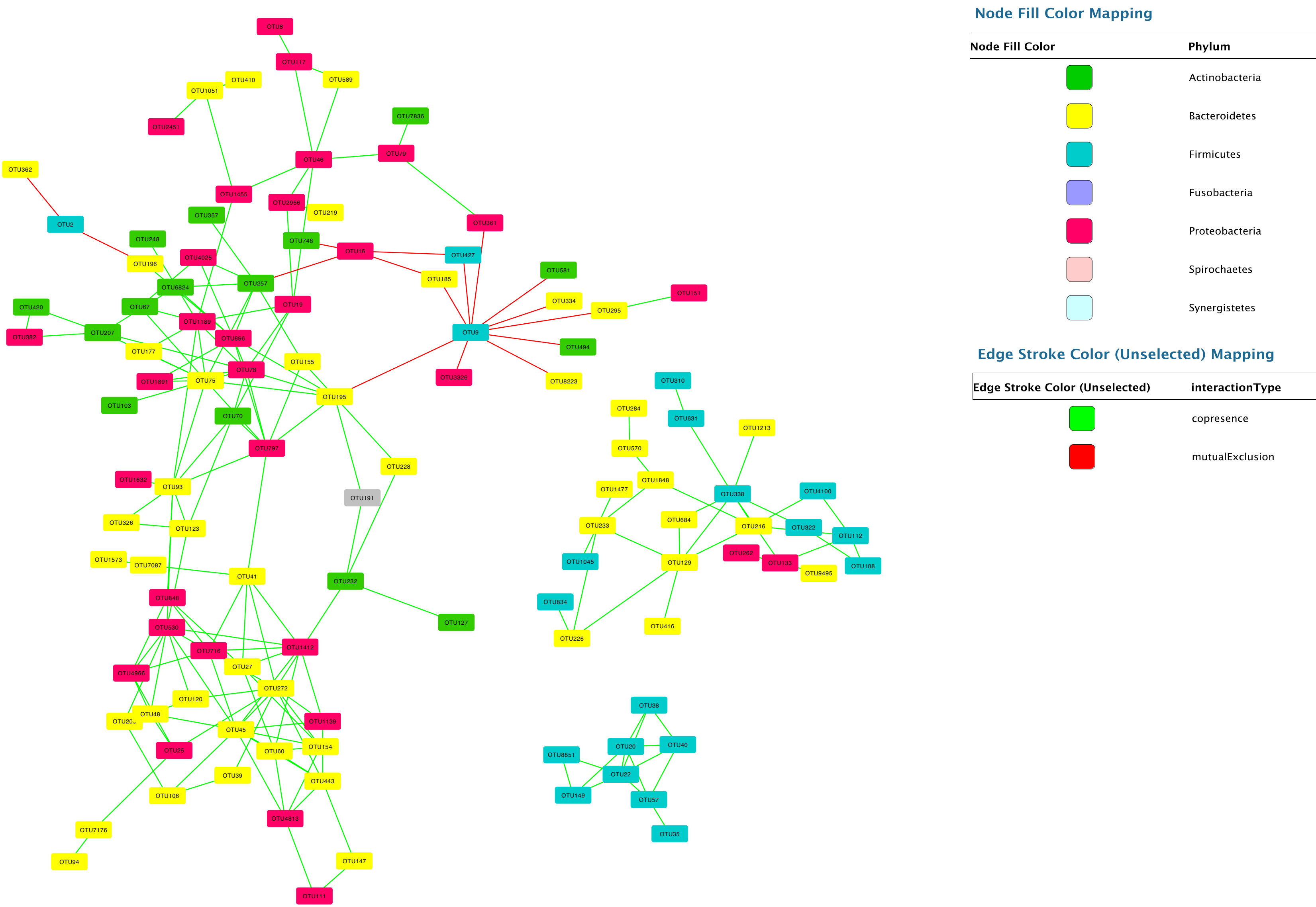

f) Day 6 intramammary microbial network for BstY(b)

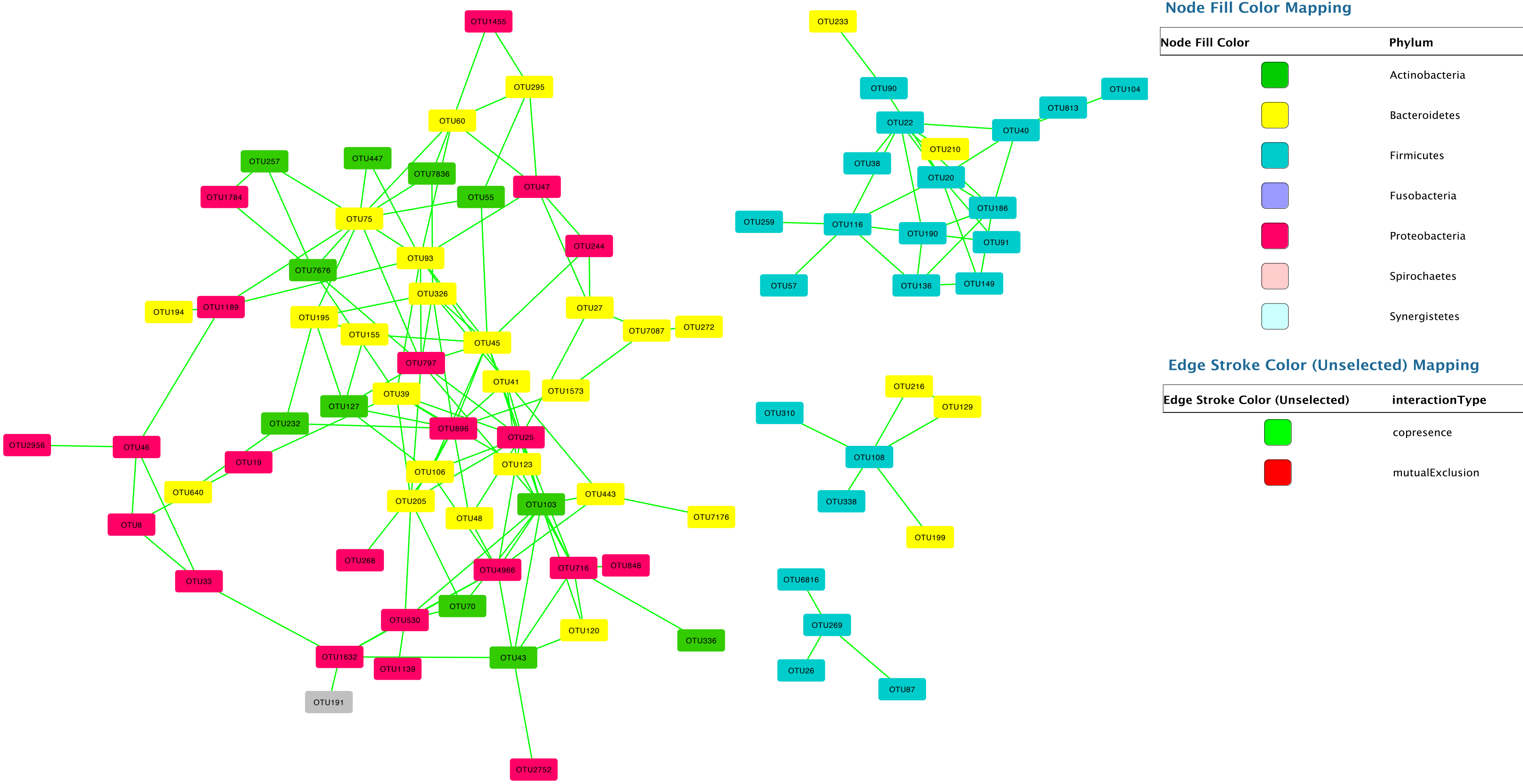

Fig S5. Bacterial co-occurrence and co-exclusion networks. Correlation network analysis (CoNet) was used to explore microbial co-occurrence/mutual-exclusion relationships. In this ensemble method, a combination of diverse measures of correlation (including Pearson's and Spearman's rank correlation coefficients) and dissimilarity (Bray-Curtis and Kullback-Leibler) were used to overcome major challenges in the inference of co-occurrence and/or co-exclusion patterns. Networks (a-f) show significant relationships (FDR corrected $q < 0.05$; supported by at least 3 methods) among bacterial OTUs within intramammary microbiota of different *BoLA-DRB3.2* variants (BstYa and BstYb) at different sampling time points (days in milk 0, 1, and 6). Nodes (representative OTUs) are colored based on originating phyla, and edges (represent significant co-occurrence/co-exclusion relationships) are colored based on the type of relationships (red = negative relationship or mutual co-exclusion and green = positive relationship or co-occurrence).

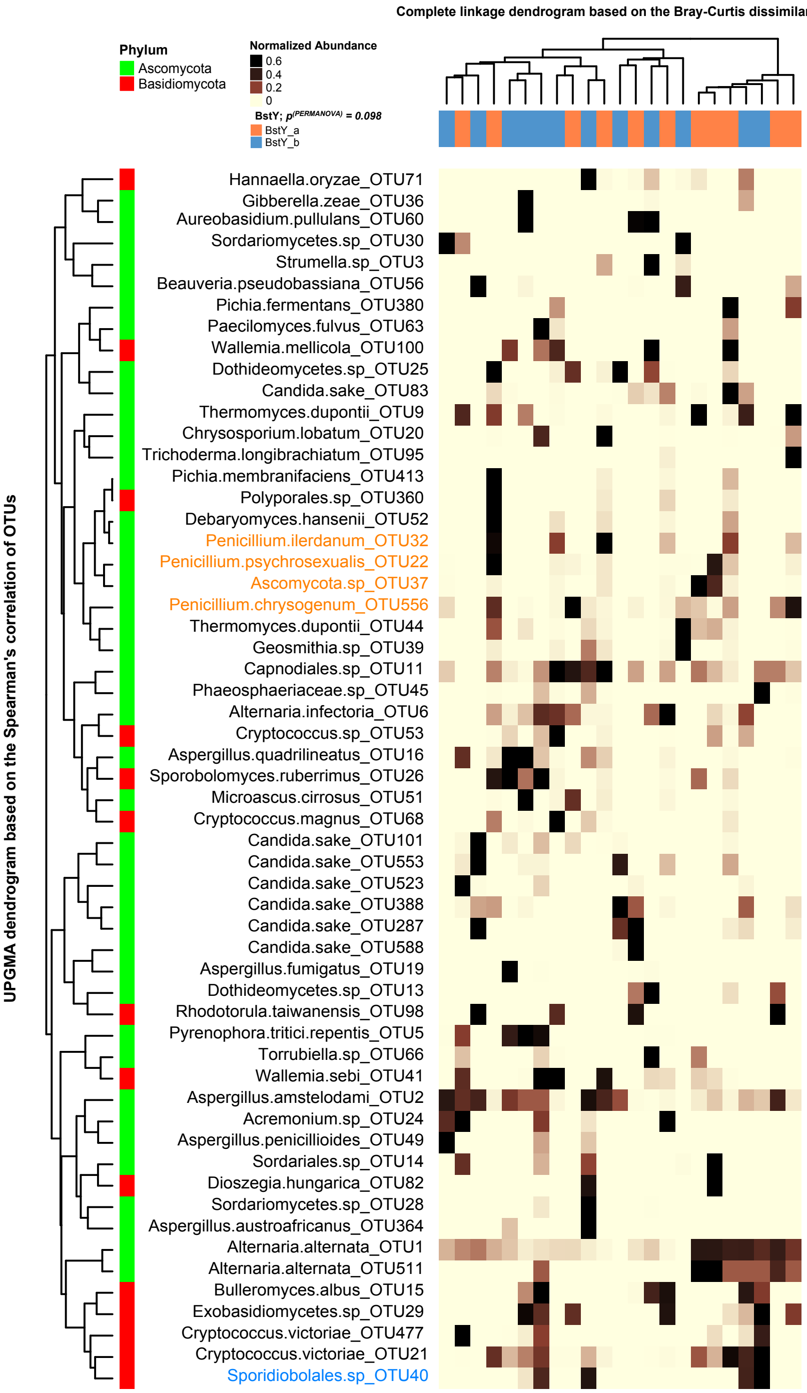

Fig S6. Unsupervised cluster analysis of day 0 colostrum samples based on the distribution of fungal OTUs. Rows correspond to individual fungal OTUs (relative abundance > 0.1% of the community). Columns correspond to individual samples, color coded based on *BoLA-DRB3.2* variants (BstYa) and (BstYb). The "Normalized Abundance" key relates colors to the normalized proportions of OTUs across samples. The top dendrogram shows the result of complete-linkage hierarchal clustering of samples based on the Bray–Curtis dissimilarities of their fungal communities. The left dendogram shows how OTUs correlate (co-occur) with each other based on their Spearman's correlation coefficient. The "Phylum" key relates the left annotations to the corresponding phylum of each OTU. Color codes have been used to highlight statistically significant associations between the proportion of OTUs and *BoLA-DRB3.2* variants (identified using Linear Discriminant Analysis Effective Size (LEfSe)). Bray-Curtis resemblance matrix was created based on the proportions of the abundant fungal OTUs and subjected to PERMANOVA (9999 permutations) in order to test the significance of the clustering pattern of samples based on *BoLA-DRB3.2* variants. A p-value < 0.05 was considered significant.

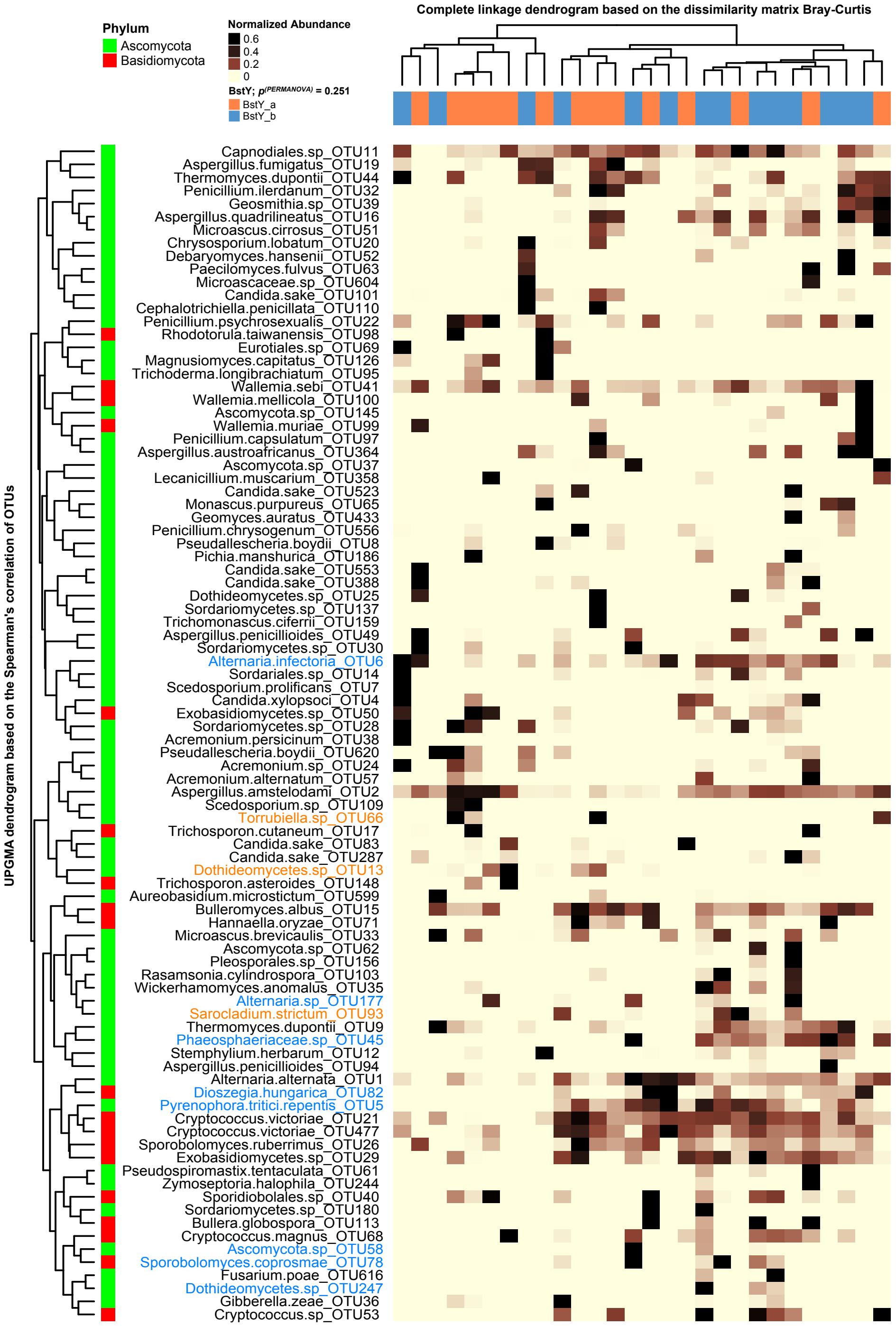

Fig S7. Unsupervised cluster analysis of day 1 colostrum samples based on the distribution of fungal OTUs. Rows correspond to individual fungal OTUs (relative abundance > 0.1% of the community). Columns correspond to individual samples, color coded based on *BoLA-DRB3.2* variants (BstYa) and (BstYb). The "Normalized Abundance" key relates colors to the normalized proportions of OTUs across samples. The top dendrogram shows the result of complete-linkage hierarchal clustering of samples based on the Bray–Curtis dissimilarities of their fungal communities. The left dendogram shows how OTUs correlate (co-occur) with each other based on their Spearman's correlation coefficient. The "Phylum" key relates the left annotations to the corresponding phylum of each OTU. Color codes have been used to highlight statistically significant associations between the proportion of OTUs and *BoLA-DRB3.2* variants (identified using Linear Discriminant Analysis Effective Size (LEfSe)). Bray-Curtis resemblance matrix was created based on the proportions of the abundant fungal OTUs and subjected to PERMANOVA (9999 permutations) in order to test the significance of the clustering pattern of samples based on *BoLA-DRB3.2* variants. A p-value < 0.05 was considered significant.

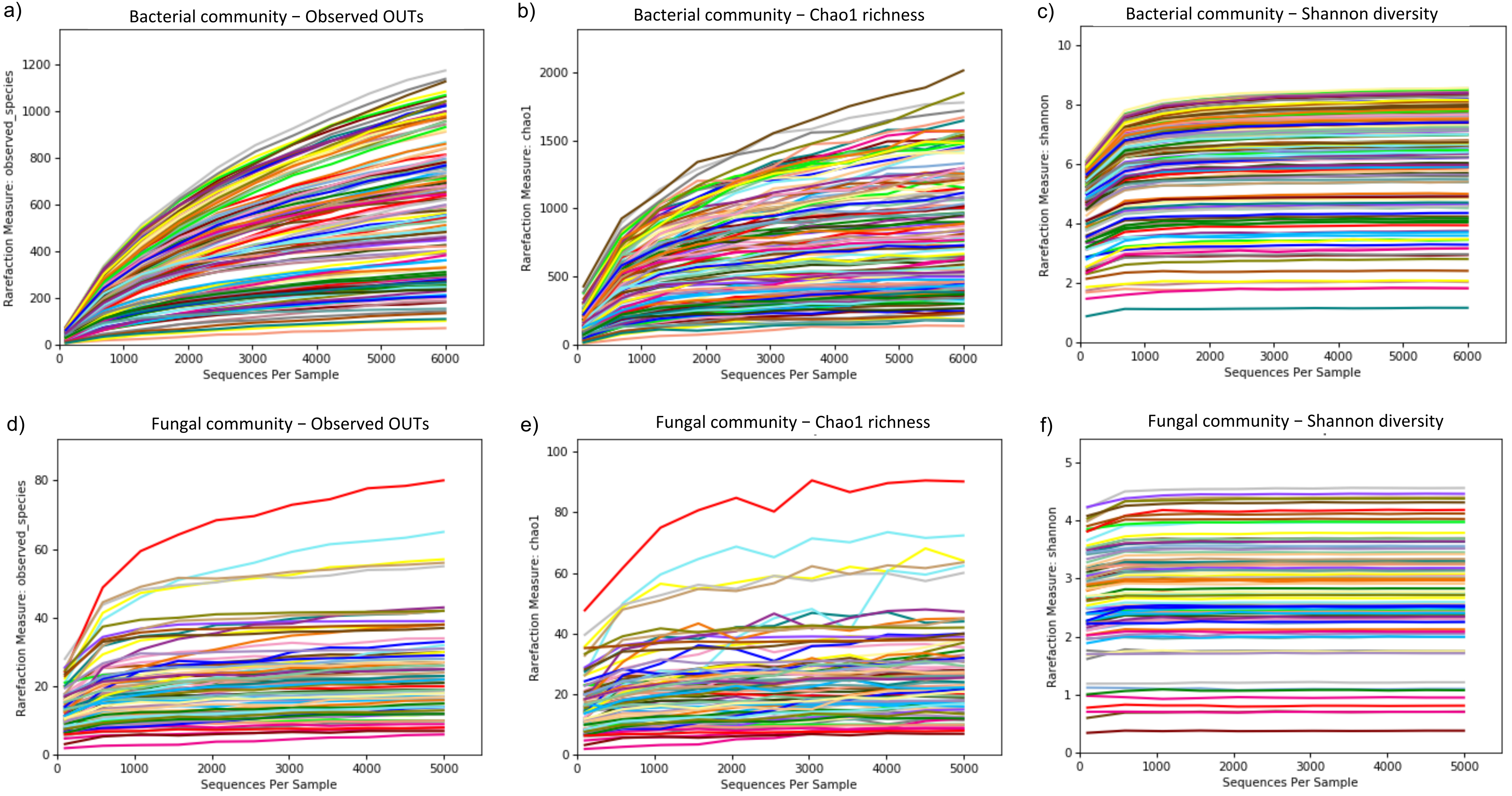

Figure S8. Rarefaction curves of of richness (Observed-OTUs and Chao1) and diversity (Shannon) indices for bacterial (a-c) and fungal (d-f) communities.
